# Supplementary material for: Hypermethylation of PRKCZ Regulated by E6 Inhibits Invasion and EMT via Cdc42 in HPV-Related Head and Neck Squamous Cell Carcinoma
Source: Cancers (Basel). 2022 Aug 27;14(17):4151. doi: 10.3390/cancers14174151 (PMC9454700; doi:10.3390/cancers14174151)
Supplement: Supplementary file 1 [file cancers-14-04151-s001.zip › cancers-1822871-supplementary.pdf]

Supplementary Material: Hypermethylation of PRKCZ Regulated by E6 Inhibits Invasion and EMT via Cdc42 in HPV-Related Head and Neck Squamous Cell Carcinoma

Hao-Fan Wang, Jian Jiang, Jia-Shun Wu, Mei Zhang, Xin Pang, Li Dai, Ya-Ling Tang * and Xin-Hua Liang *

Table S1. The top 25 abnormally hypermethylated genes in HPV+ HNSCC tissue compared with HPV- HNSCC tissue.

Gene Name	Target ID	case. AVG_Beta	case. DiffScore	case. Delta Beta	case. Detection Pval	control. AVG_Beta	control. Detection Pval
ASCL4	cg20443254	0.294272	48.16483	0.218263	0	0.076009	0
NEFM	cg07502389	0.291245	44.35434	0.193643	0	0.097601	0
LINGO3	cg19241679	0.305958	38.99714	0.189708	0	0.11625	0
NLRC4	cg22805603	0.714048	38.6867	0.232118	0	0.48193	0
CLEC4GP1	cg24889914	0.46479	38.30906	0.203835	0	0.260955	0
LRP1B	cg00603435	0.624908	37.18912	0.206679	0	0.418229	0
SCOC	cg13913247	0.821391	32.91441	0.200871	0	0.62052	0
PTGFR	cg27046936	0.361525	32.68072	0.247227	0	0.114298	0
ZIC2	cg24690071	0.356628	32.35521	0.19429	0	0.162338	0
GLRA1	cg12995800	0.237169	28.96347	0.190496	0	0.046672	0
TBX18	cg07028914	0.346965	28.67912	0.257072	0	0.089893	0
GJB6	cg07610777	0.557075	28.15817	0.247783	0	0.309292	0
MAPT	cg18228076	0.576034	28.09607	0.254003	0	0.322031	0
LYNX1	cg04688828	0.247635	28.07237	0.191953	0	0.055682	0
CCDC3	cg03540175	0.430408	27.85628	0.184329	0	0.246079	0
C2orf39	cg04726446	0.266014	27.42363	0.177761	0	0.088253	0
SYCN	cg02863073	0.241393	26.401	0.189945	0	0.051448	0
C1orf106	cg00909514	0.256152	26.32339	0.236464	0	0.019688	0
SNTG1	cg14291467	0.661534	25.96148	0.254655	0	0.406879	0
PRKCZ	cg06157219	0.405768	25.67634	0.198964	0	0.206804	0
ZFP37	cg03454353	0.349559	25.53246	0.172653	0	0.176906	0
ACCN4	cg19210770	0.266877	25.50808	0.179662	0	0.087215	0
CLEC4D	cg01253753	0.613357	25.28931	0.191497	0	0.42186	0
PDZRN3	cg14473924	0.439253	24.9945	0.242738	0	0.196515	0
DTNA	cg02790691	0.673311	24.91104	0.190286	0	0.483026	0

Table S2. The top 25 abnormally hypomethylated genes in HPV+ HNSCC tissue compared with HPV- HNSCC tissue.

Gene Name	Target ID	case. AVG_Beta	case. DiffScore	case. Delta Beta	case. Detection Pval	control. AVG_Beta	control. Detection Pval
NR2F1	cg08969950	0.329318	-19.1478	-0.20643	0	0.53575	0
CNGA3	cg22241124	0.519476	-19.5996	-0.19182	0	0.7113	0
EBF2	cg16895719	0.386037	-19.7555	-0.17668	0	0.562714	0
GLI3	cg18100008	0.606943	-19.8773	-0.17067	0	0.777615	0
LRRN4	cg19097280	0.581911	-19.936	-0.18212	0	0.764033	0
HOXD8	cg03321133	0.291694	-20.2312	-0.19908	0	0.490772	0
OR9Q1	cg05922911	0.677669	-20.2602	-0.18958	0	0.867245	0
DLX1	cg15552158	0.330394	-21.3701	-0.2068	0	0.537197	0
SOX9	cg11350586	0.381243	-21.4671	-0.22397	0	0.605209	0
TMEM232	cg06414816	0.441568	-21.829	-0.1919	0	0.633469	0
BOC	cg07481097	0.492096	-22.1367	-0.18447	0	0.676565	0
RUNX3	cg00147638	0.601542	-22.6817	-0.20773	0	0.809276	0
LHX8	cg09222749	0.231537	-23.1813	-0.17586	0	0.407392	0
DMRT2	cg00934355	0.365495	-23.6385	-0.21379	0	0.579286	0
HOXD9	cg09173768	0.417908	-23.6387	-0.27434	0	0.692252	0
HOXD3	cg00005847	0.320798	-23.7948	-0.28368	0	0.604473	0
PTCHD3	cg04500084	0.564269	-25.2987	-0.21899	0	0.783258	0
SECTM1	cg27666046	0.462912	-26.6919	-0.17257	0	0.635485	0
SLC38A1	cg17090968	0.340748	-29.6816	-0.31124	0	0.651988	0
FHIT	cg21541638	0.337263	-31.1058	-0.22638	0	0.56364	0
SP9	cg00928397	0.27409	-31.5759	-0.20606	0	0.480155	0
POU4F1	cg06675375	0.185358	-32.8697	-0.19039	0	0.375743	0
HOXD4	cg12127282	0.44339	-36.5882	-0.25385	0	0.697236	0
AXIN2	cg17797591	0.325205	-40.421	-0.24282	0	0.568027	0
MACROD2	cg09937190	0.52854	-42.1664	-0.27296	0	0.801501	0

Table S3. Clinicopathological Characteristics of Patients whose tissue specimens were used for qMSP.

Parameters	HPV+	HPV-
	Number of cases	Number of cases
Age		
＜60	4	14
＞60	6	16
Gender		
Male	7	21
Female	3	9
T stage		
T1	2	8
T2	3	11
T3	5	7
T4	0	4
Pathological grade		
G1	2	16
G2	1	10
G3	7	4
Metastasis		
Yes	3	9
No	7	21
Recurrence		
Yes	3	5
No	7	25

Table S4. Clinicopathological Characteristics of Patients whose tissue specimens were used for array.

	Case 1	Case 3	Case 4	Case 5	Case B	Case C
Age	66	68	44	58	78	61
Gender	Male	Male	Male	Male	Male	Male
T stage	4	3	3	4	2	2
Pathological grade	III	II	I	I	I	II
Metastasis	Yes	Yes	No	No	No	Yes
Recurrence	Yes	No	No	No	No	Yes
HPV status	Positive	Positive	Positive	Negative	Negative	Negative

Table S5. Primers of genes for qRT-PCR.

Gene	Forward primer	Reverse primer
GAPDH	TGGCCAAGGTCATCCATGAC	TGTCATACCAGGAAATGAGCTTG
PRKCZ	AAATCTCTCAGGGGCTTGG	TCACCACTTTCATGGCGTA
HPV-E6	GAGAACTGCAATGTTTCAGGACC	TGTATAGTTGTTTGCAGCTCTGTGC
HPV-E7	CGGAATTCATGCATGGAGATACACCTACAT	CGGGAAGCTTATGGTTTCTGAGAACAGATGG
DNMT1	TACCTGGACGACCCTGACCTC	CGTTGGCATCAAAGATGGACA
DNMT3a	TATTGATGAGCGCACAAGAGAGC	GGGTGTTCCAGGGTAACATTGAG
DNMT3b	GGCAAGTTCTCCGAGGTCTCTG	TGGTACATGGCTTTTCGATAGGA
Rap1A	CGTGAGTACAAGCTAGTGGTCC	CCAGGATTTCGAGCATACACTG
Rap1B	AACAGATTCTTCGAGTTAAAGACACTGA	TTTGACCTTGTTCCTTCCCTACA
Rac1	ATAGGCCCAGATTCACTGGTT	GAGACGGAGCTGTTGGTAAAA
Cdc42	CAAGGACATTTGTTTGCCATT	TTTGGTGCATTTCAAAGGTG
RhoA	TATCGAGGTGGATGGAAAGC	TTCTGGGGTCCACTTTTCTG
RhoB	TTGTGCCTGTCCTAGAAGTG	CAAGTGTGGTCAGAATGCTAC
Epac1	CCTCTCCAACTCGGTGAAGC	CTGGCTGAACAACACGGTC
E-cad	ACATACACTCTCTTCTCTC	GTCATTCTGATCGGTTAC
N-cad	ATCATCCTGCTTATCCTT	TTATCTCTTACATCATCTTCTG
Vimentin	CTGAGGGAAACTAATCTG	TTGATAACCTGTCCATCT

Table S6. SiRNA sequences of genes.

Gene	Sequence（5’-3’）
E6 siRNA-2	GGUCGAUGUAUGUCUUGUUTTAACAAGACAUACAUCGACCTT
E7 siRNA-3	UAACAGUAGAGAUCAGUUGUCCAACUGAUCU-CUACUGUUAUG
PRKCZ siRNA-1	GCAAACUGCUGGUCCAUAATT UUAUGGACCAGCAGUUUGCTT
PRKCZ siRNA-2	CCGGAAGCAUGACAGCAUUTT AAUGCUGUCAUGCUUCCGGTT
Control siRNA	UUCUCCGAACGUGUCACGUTTACGUGACACGUUCGGAGAATT

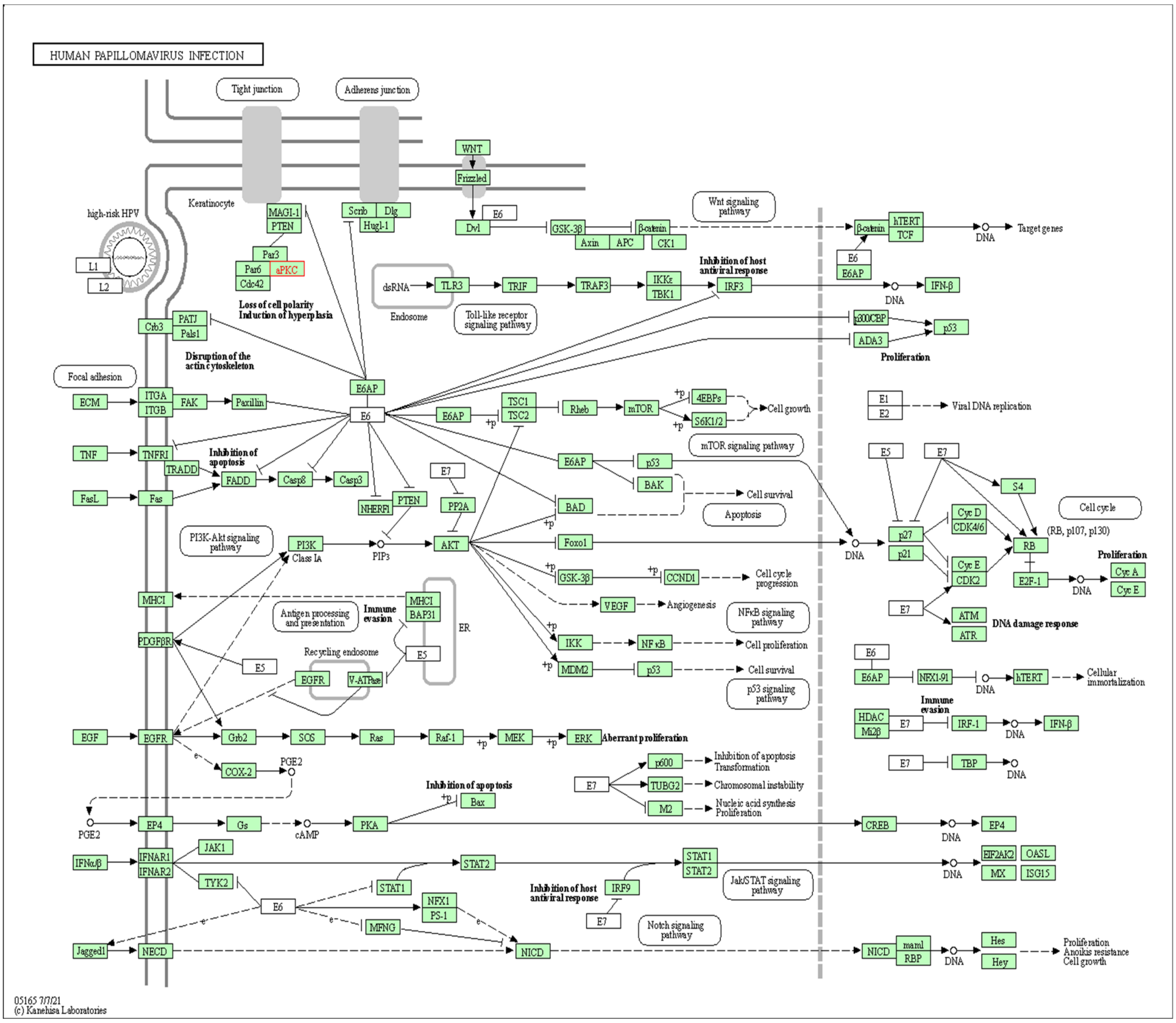

Figure S1. The KEGG pathways of Human papillomavirus infection.

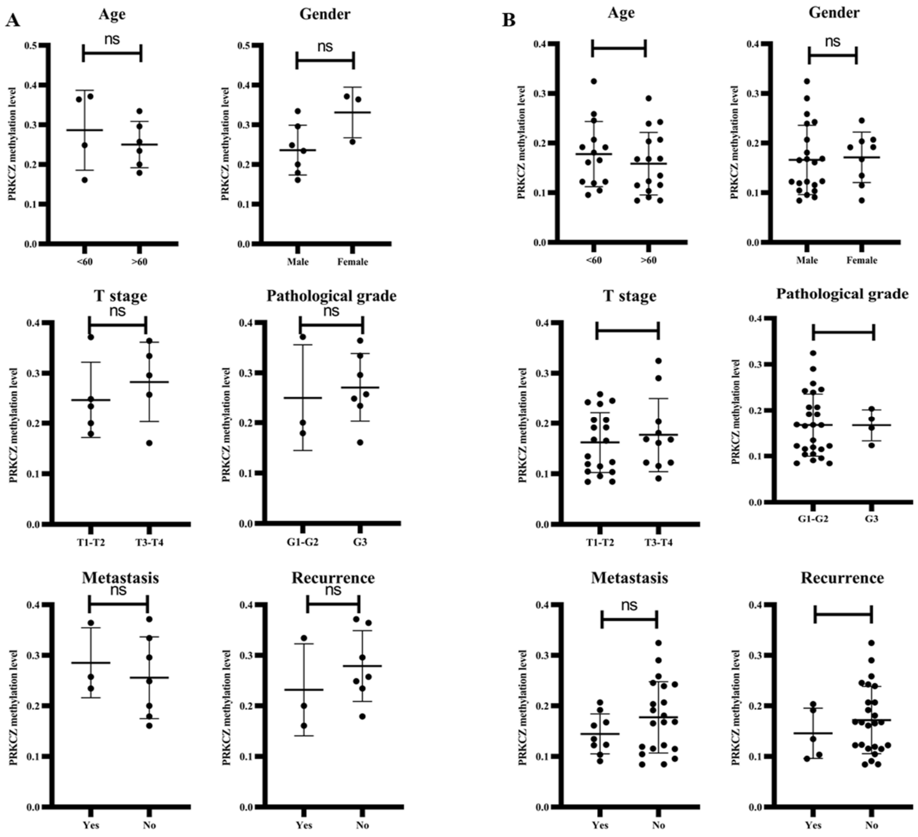

Figure S2. The relations between PRKCZ methylation status and relevant clinical and pathological parameters of HPV+ and HPV- patients.

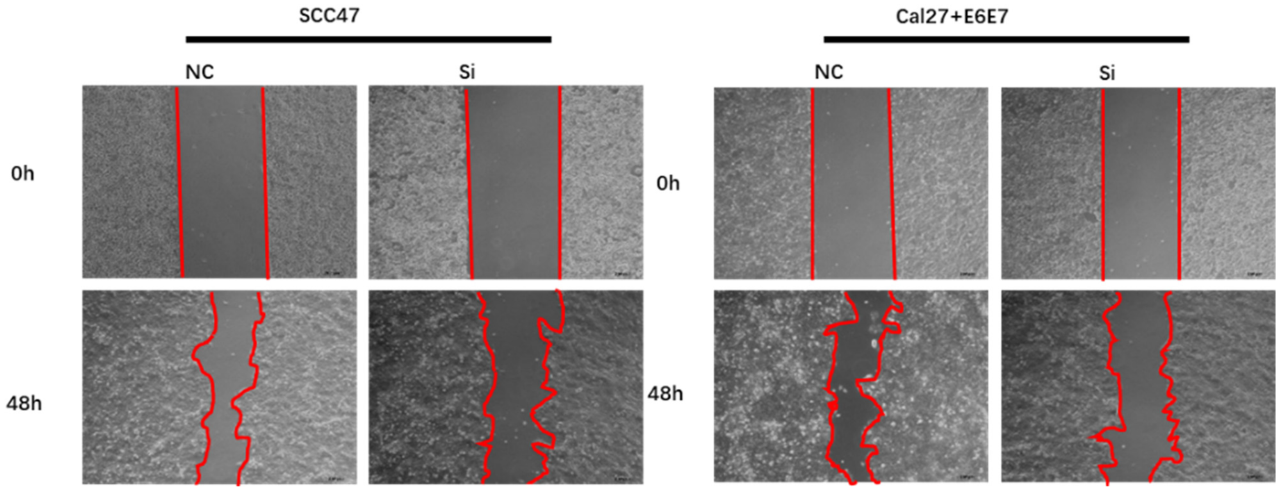

Figure S3. The results of wound healing assays between PRKCZ siRNA and control siRNA groups. The results showed that the wounding healing areas of HPV+ HNSCC cells transfected with PRKCZ siRNA were little than that of transfected with control siRNA.

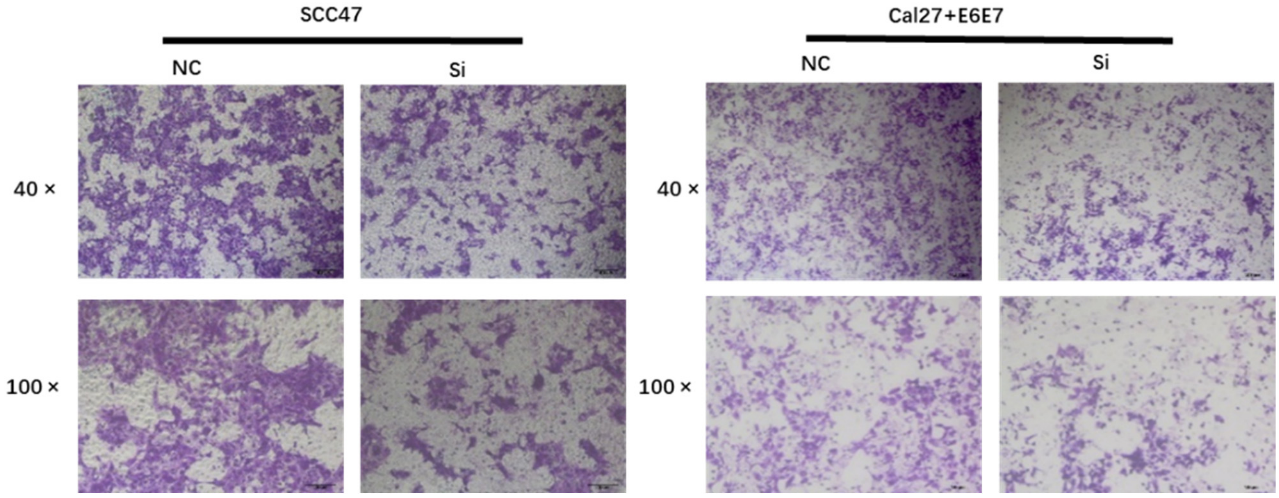

Figure S4. The results of transwell invasion assays between PRKCZ siRNA and control siRNA groups. The results showed that the invade cells number of HPV+ HNSCC cells transfected with PRKCZ siRNA were smaller than that of transfected with control siRNA.

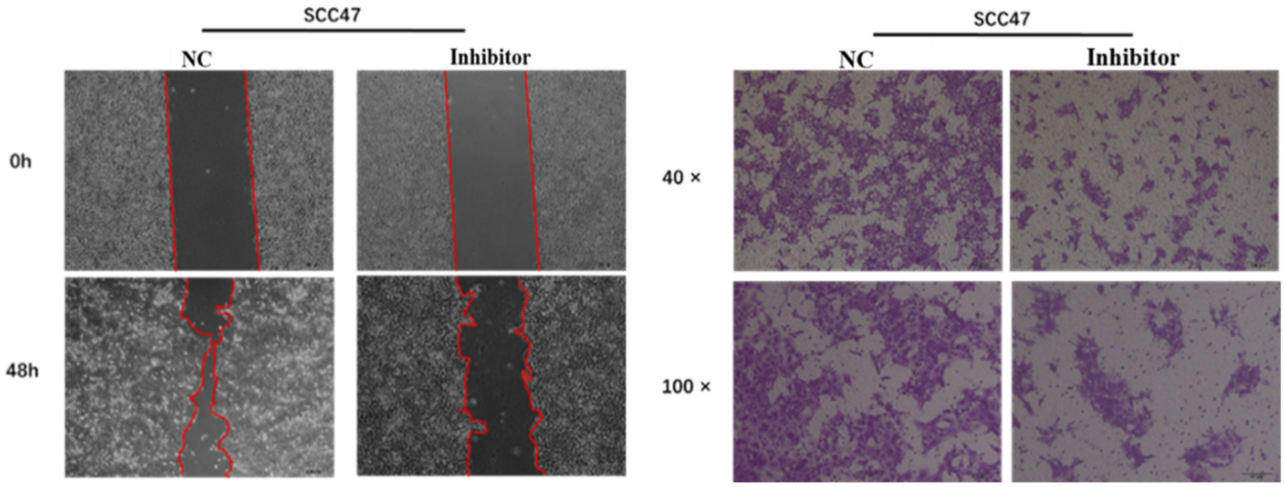

Figure S5. The results of wound healing and transwell invasion assays between pseudosubstrate inhibitor and normal groups. The results showed that both the wounding healing areas and the invade cells number of SCC47 transfected with pseudosubstrate inhibitor were smaller than that of normal SCC47.

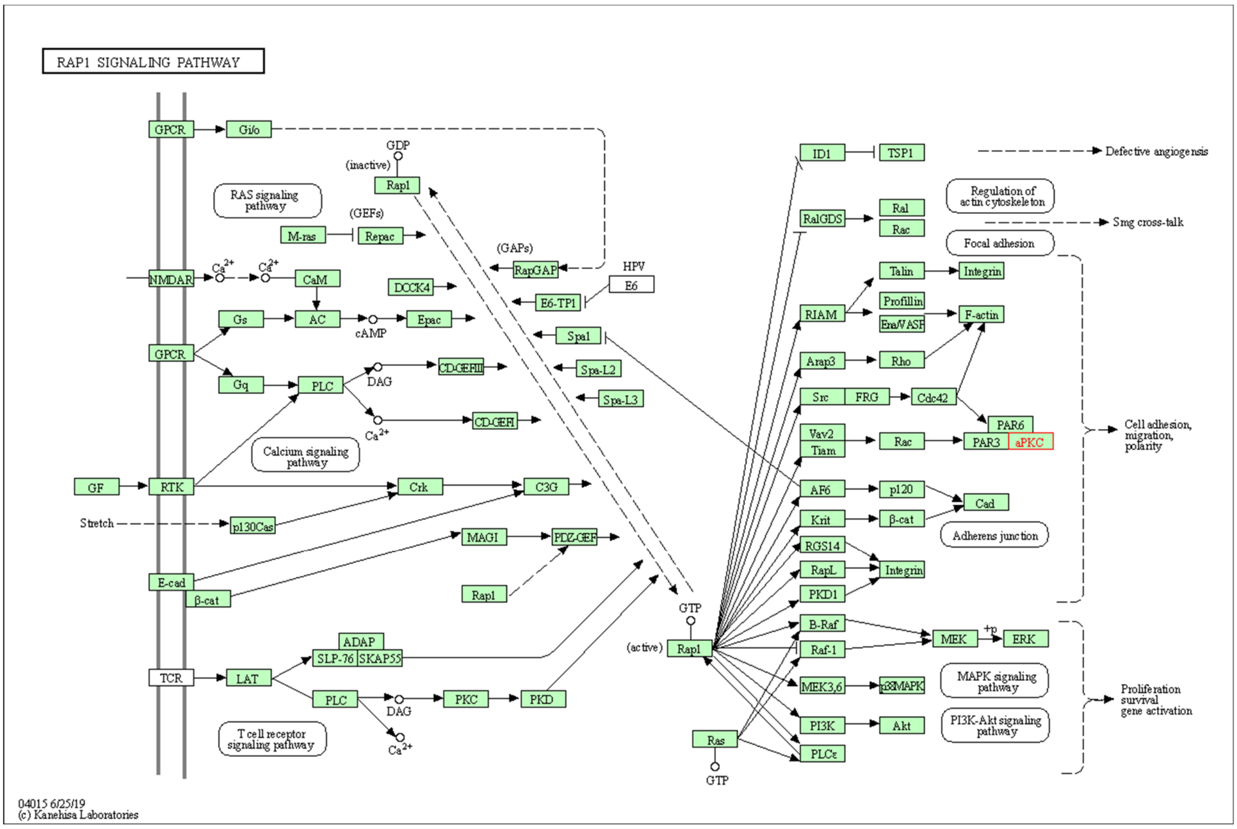

Figure S6. The KEGG pathways of RAP1 signaling pathway.
