# Supplementary material for: Hypermethylation of PRKCZ Regulated by E6 Inhibits Invasion and EMT via Cdc42 in HPV-Related Head and Neck Squamous Cell Carcinoma
Source: Cancers (Basel). 2022 Aug 27;14(17):4151. doi: 10.3390/cancers14174151 (PMC9454700; doi:10.3390/cancers14174151)
Supplement: Supplementary file 1 [file cancers-14-04151-s001.zip › File S1/The densitometry scans of each WB figure.pdf]

The densitometry scans of each WB figure

Figure 2B PKC-ζ	SCC090	SCC47	Cal27	SCC25	SCC9
densitometry readings	22675.196	21401.024	26081.489	33437.803	29158.853

Figure 2D PKC-ζ	0uM	2.5uM	5uM	10uM
densitometry readings	16112.752	19221.137	23306.258	36206.066

Figure 2F PKC-ζ	0uM	2.5uM	5uM	10uM
densitometry readings	16091.116	18844.995	23051.874	28825.752

Figure 3A E6	NC	E6E7
densitometry readings	75765.070	7114.886

Figure 3A E7	NC	E6E7
densitometry readings	84038.371	5694.690

Figure 3B PKC-ζ SCC47	NC	Si-1	Si-2
densitometry readings	43611.108	19217.108	24387.380

Figure 3B PKC-ζ Cal27+E6E7	NC	Si-1	Si-2
densitometry readings	45924.794	25272.723	28010.652

Figure 3G PKC-ζ	NC	Inhibitor
densitometry readings	80983.655	9182.865

Figure 4B E6	NC	E6-Si1	E6-Si2	E6-Si3
densitometry readings	40910.551	27309.288	15009.288	4991.004

Figure 4B E7	NC	E7-Si1	E7-Si2	E7-Si3
densitometry readings	31547.844	21709.388	10385.459	13355.510

Figure 4E DNMT1	NC	si-E6	si-E7
densitometry readings	52732.108	36633.673	42763.229

Figure 4E DNMT3a	NC	si-E6	si-E7
densitometry readings	55558.652	44572.158	41140.350

Figure 4E DNMT3b	NC	si-E6	si-E7
densitometry readings	49426.593	36334.823	44371.765

Figure 5C PKC-ζ SCC47	NC	Si
densitometry readings	59288.090	29871.312

Figure 5C Cdc42 SCC47	NC	Si
densitometry readings	51025.111	22605.392

Figure 5C PKC-ζ Cal27+E6E7	NC	Si
densitometry readings	50535.614	233941.828

Figure 5C Cdc42 Cal27+E6E7	NC	Si
densitometry readings	49249.475	21817.907

Figure 5D E-cad SCC47	NC	Si
densitometry readings	68553.894	81476.593

Figure 5D N-cad SCC47	NC	Si
densitometry readings	71276.149	37425.655

Figure 5D Vim SCC47	NC	Si
densitometry readings	47936.279	240568.247

Figure 5D E-cad Cal27+E6E7	NC	Si
densitometry readings	54220.966	76017.907

Figure 5D N-cad Cal27+E6E7	NC	Si
densitometry readings	65078.785	35048.371

Figure 5D Vim Cal27+E6E7	NC	Si
densitometry readings	70724.777	54478.505
