# Supplementary figures and images for: Hypermethylation of PRKCZ Regulated by E6 Inhibits Invasion and EMT via Cdc42 in HPV-Related Head and Neck Squamous Cell Carcinoma
Source: Cancers (Basel). 2022 Aug 27;14(17):4151. doi: 10.3390/cancers14174151 (PMC9454700; doi:10.3390/cancers14174151)

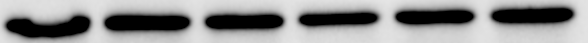

Supplement: Supplementary file 1 [file cancers-14-04151-s001.zip › File S1/Figure S2/Figure S2B Gapdh.tif]

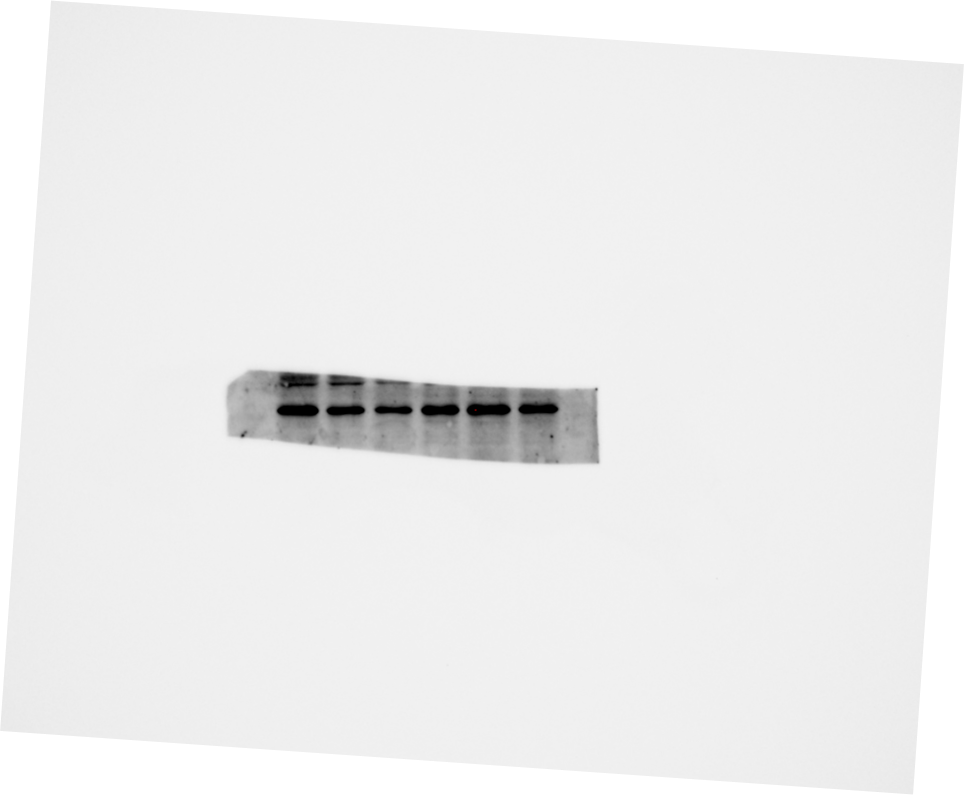

Supplement: Supplementary file 1 [file cancers-14-04151-s001.zip › File S1/Figure S2/Figure S2B PRKCZ.tif]

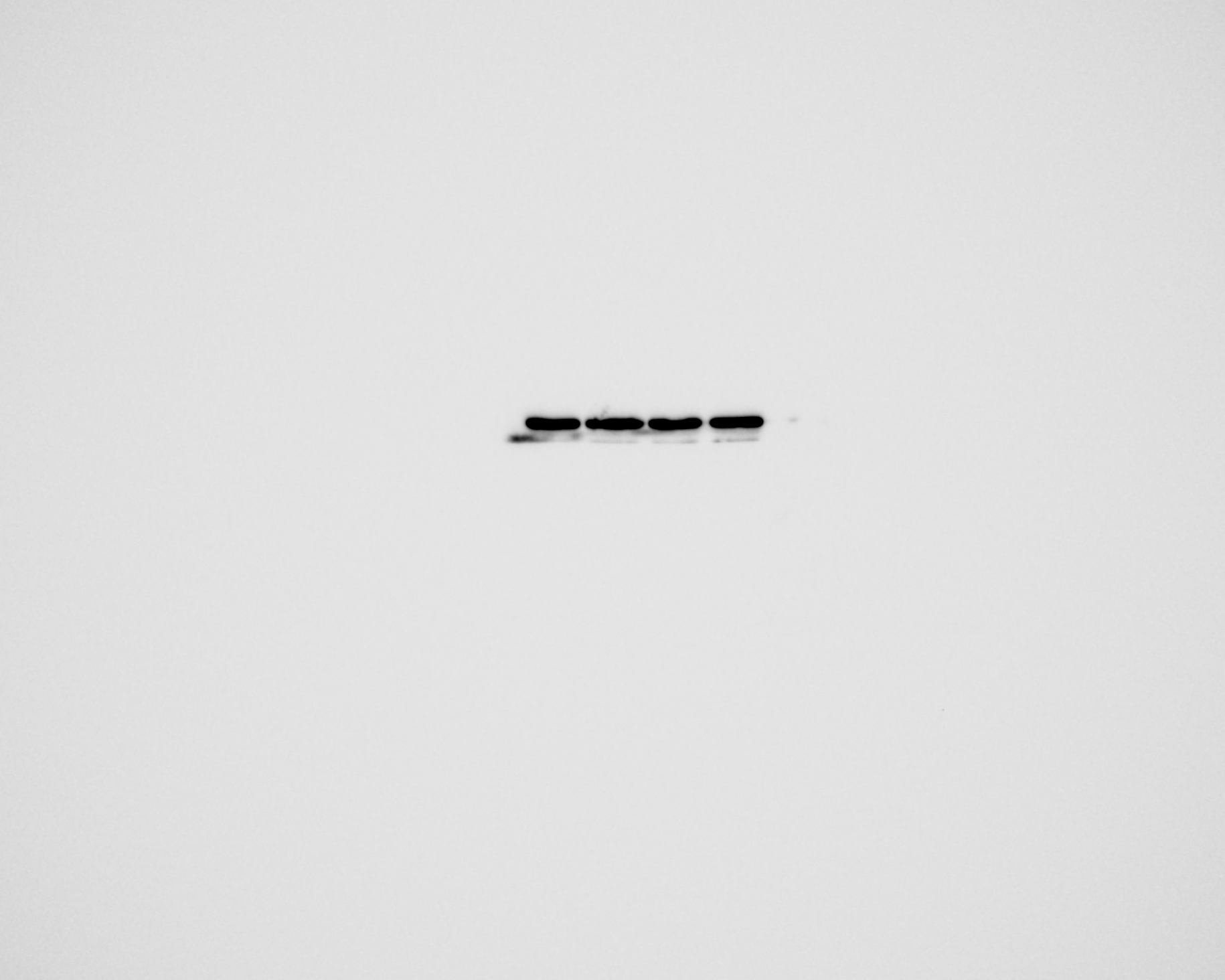

Supplement: Supplementary file 1 [file cancers-14-04151-s001.zip › File S1/Figure S2/FigureS2D SCC47 Gapdh.tif]

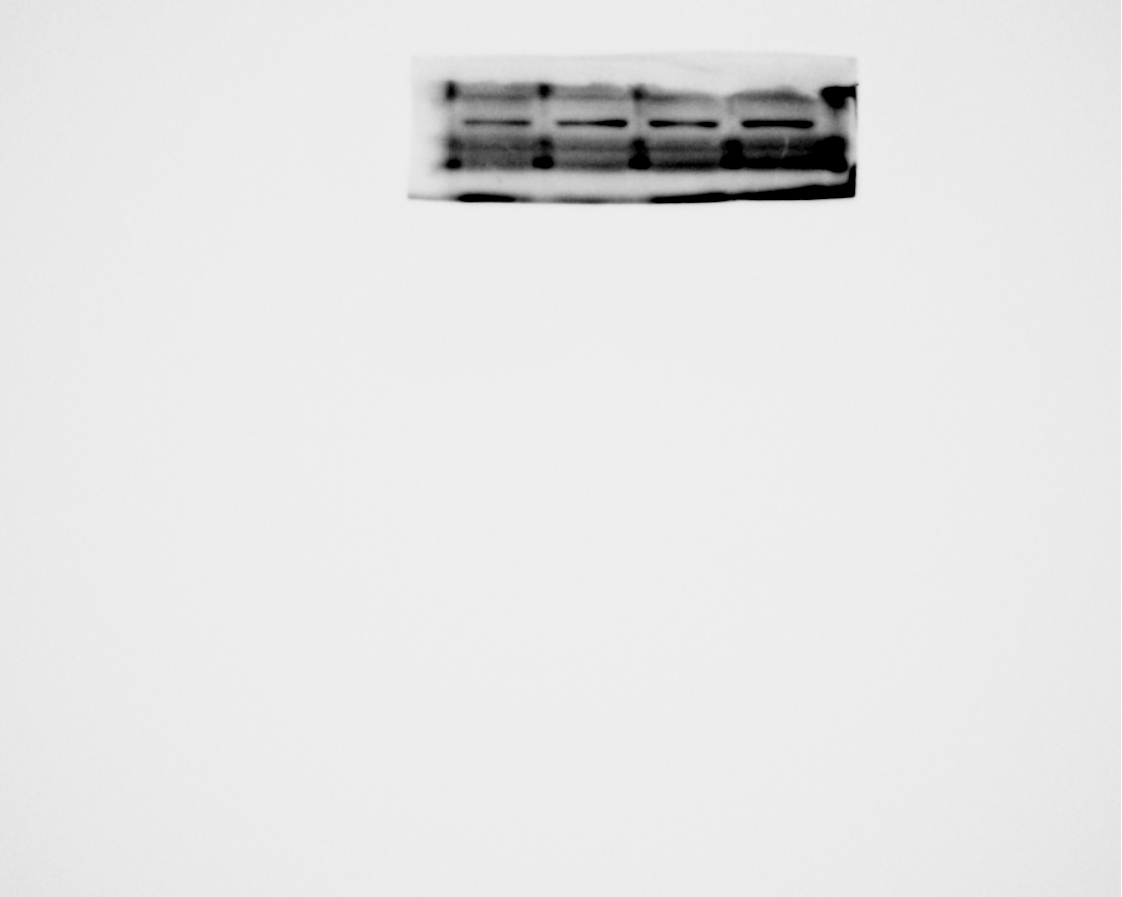

Supplement: Supplementary file 1 [file cancers-14-04151-s001.zip › File S1/Figure S2/FigureS2D SCC47 PRKCZ.jpg]

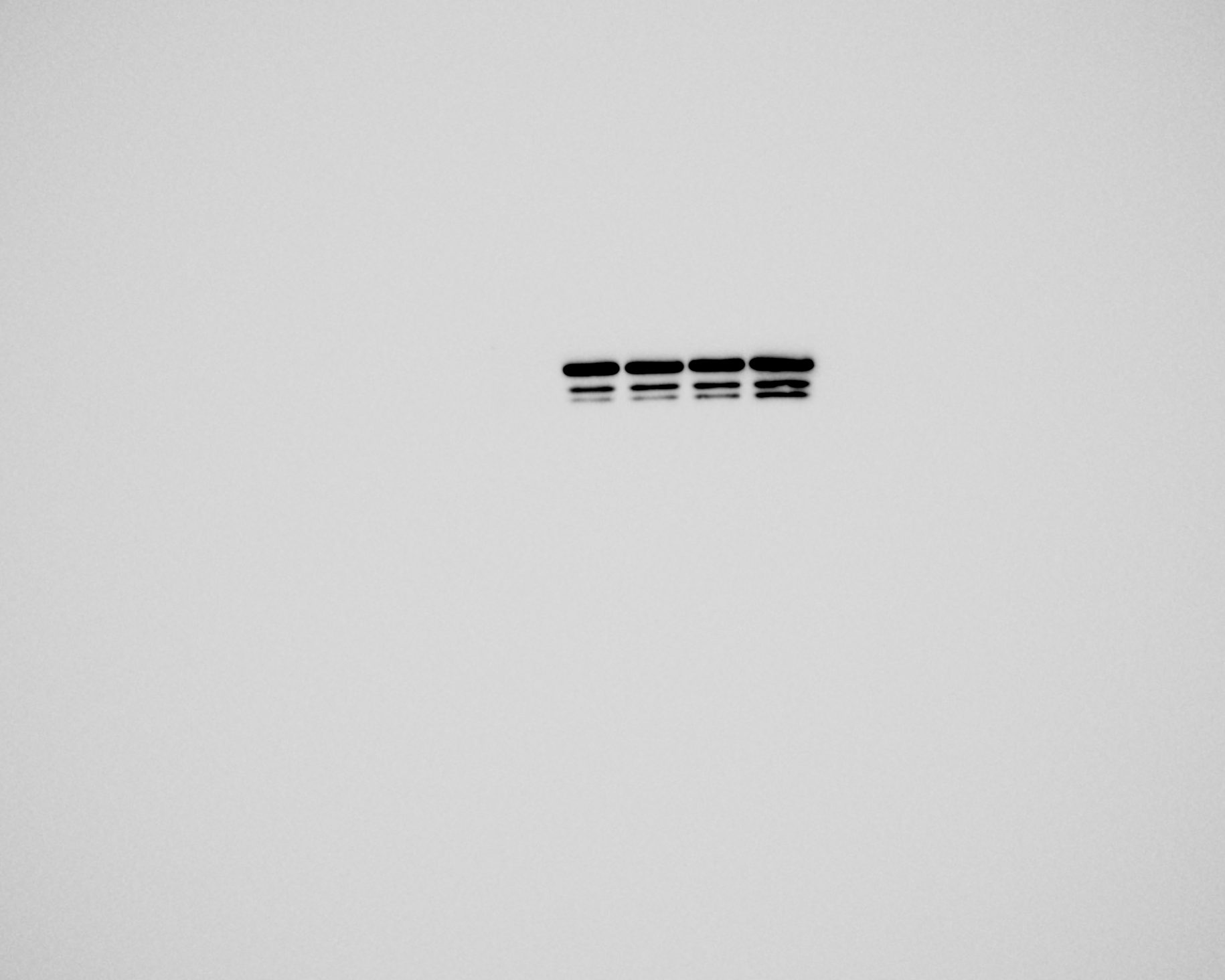

Supplement: Supplementary file 1 [file cancers-14-04151-s001.zip › File S1/Figure S2/FigureS2F Cal27 Gapdh.tif]

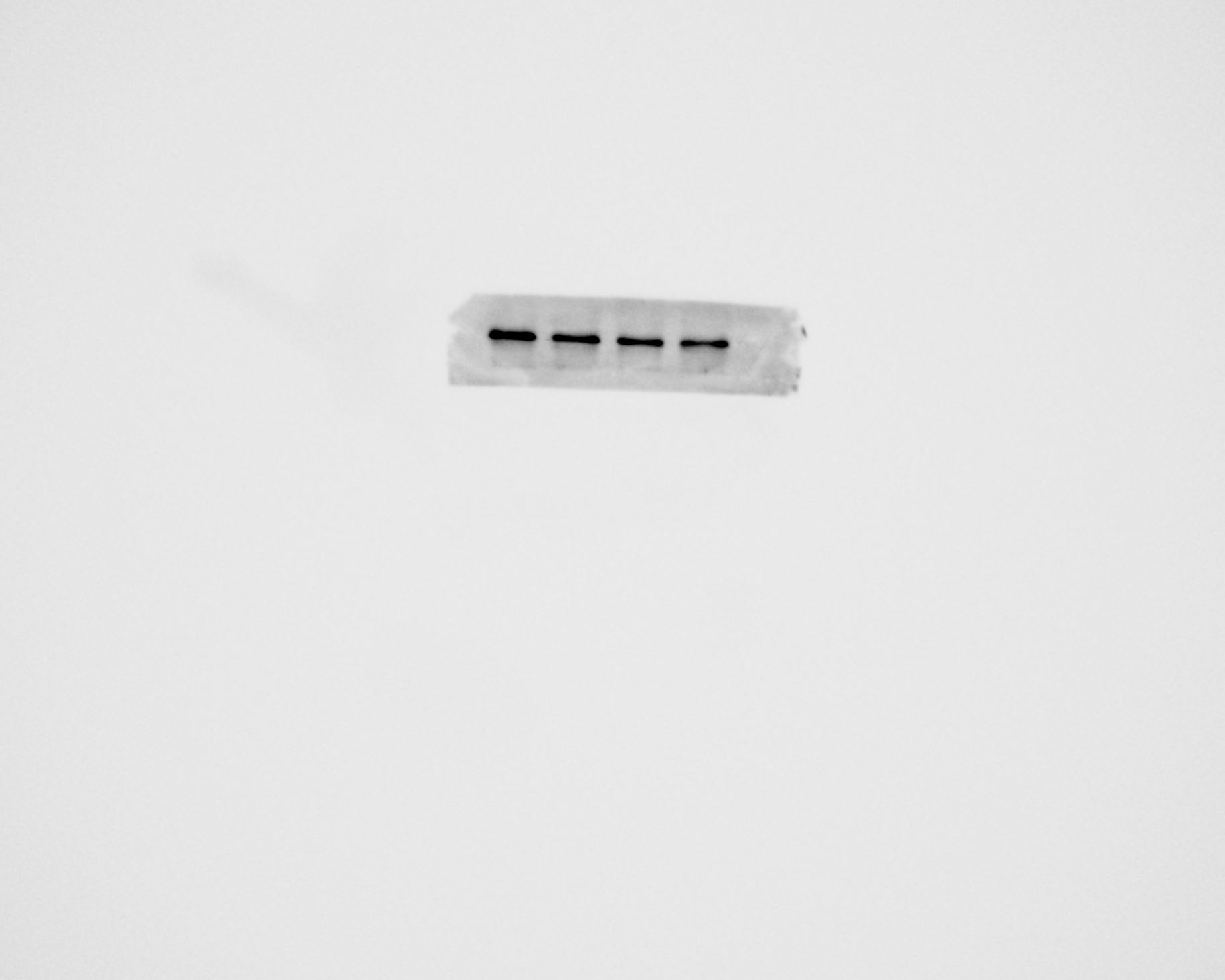

Supplement: Supplementary file 1 [file cancers-14-04151-s001.zip › File S1/Figure S2/FigureS2F Cal27 PRKCZ.tif]

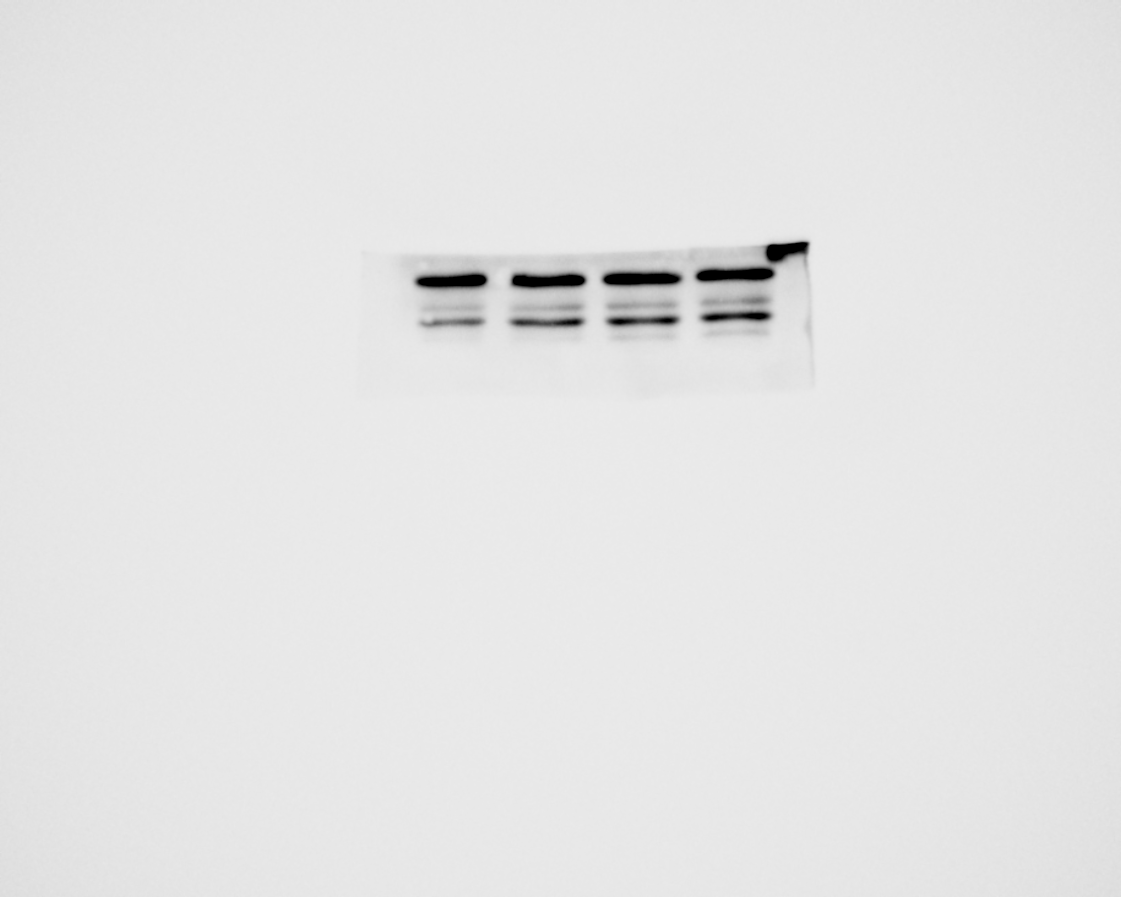

Supplement: Supplementary file 1 [file cancers-14-04151-s001.zip › File S1/Figure S3/FigerS3A gapdh.tif]

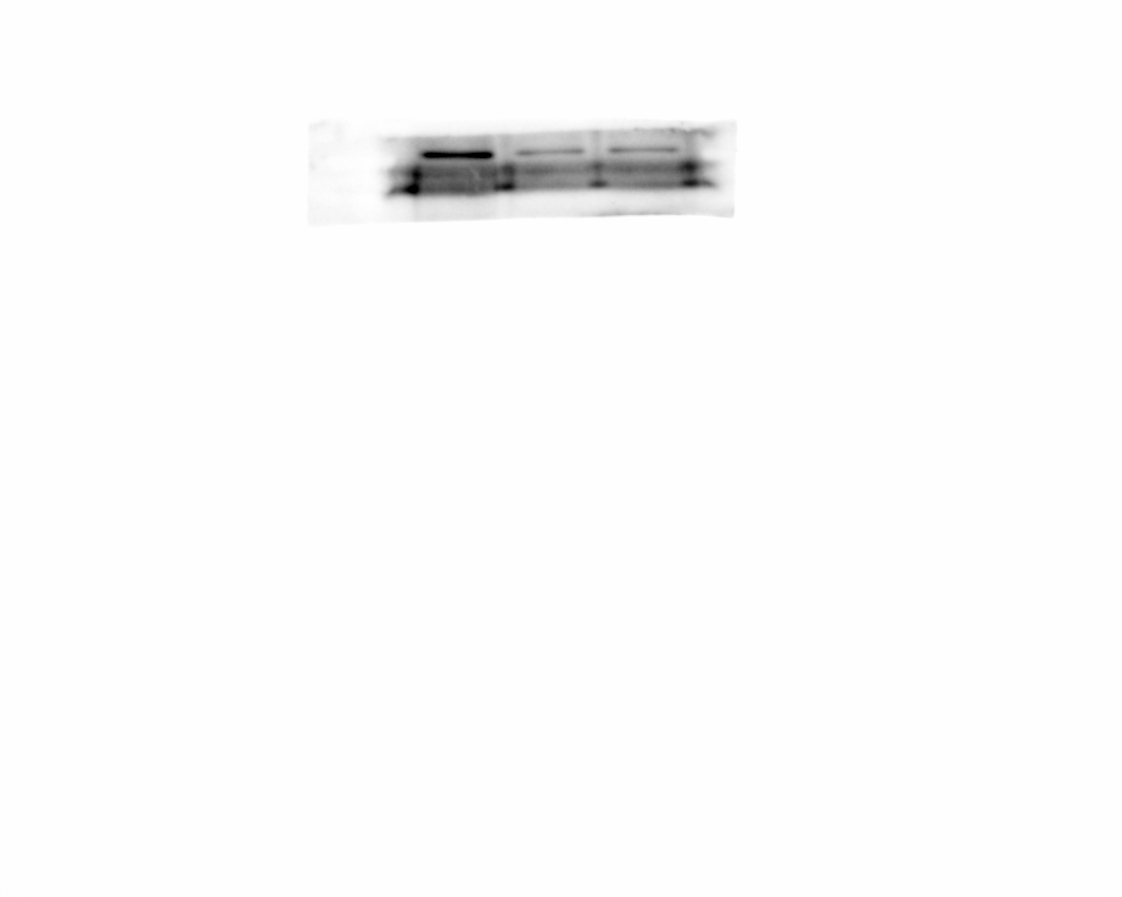

Supplement: Supplementary file 1 [file cancers-14-04151-s001.zip › File S1/Figure S3/FigerS3A E6.jpg]

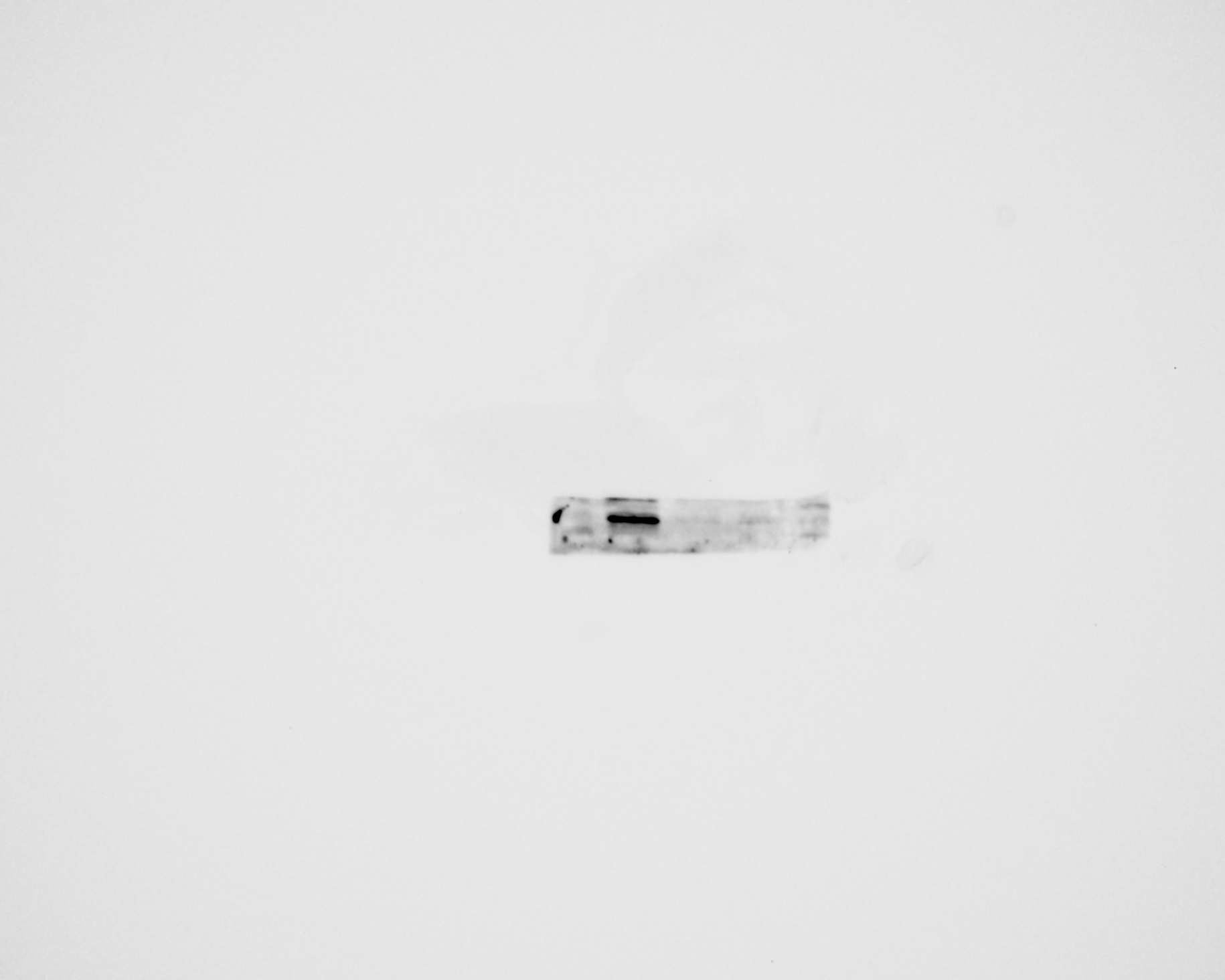

Supplement: Supplementary file 1 [file cancers-14-04151-s001.zip › File S1/Figure S3/FigerS3A E7.tif]

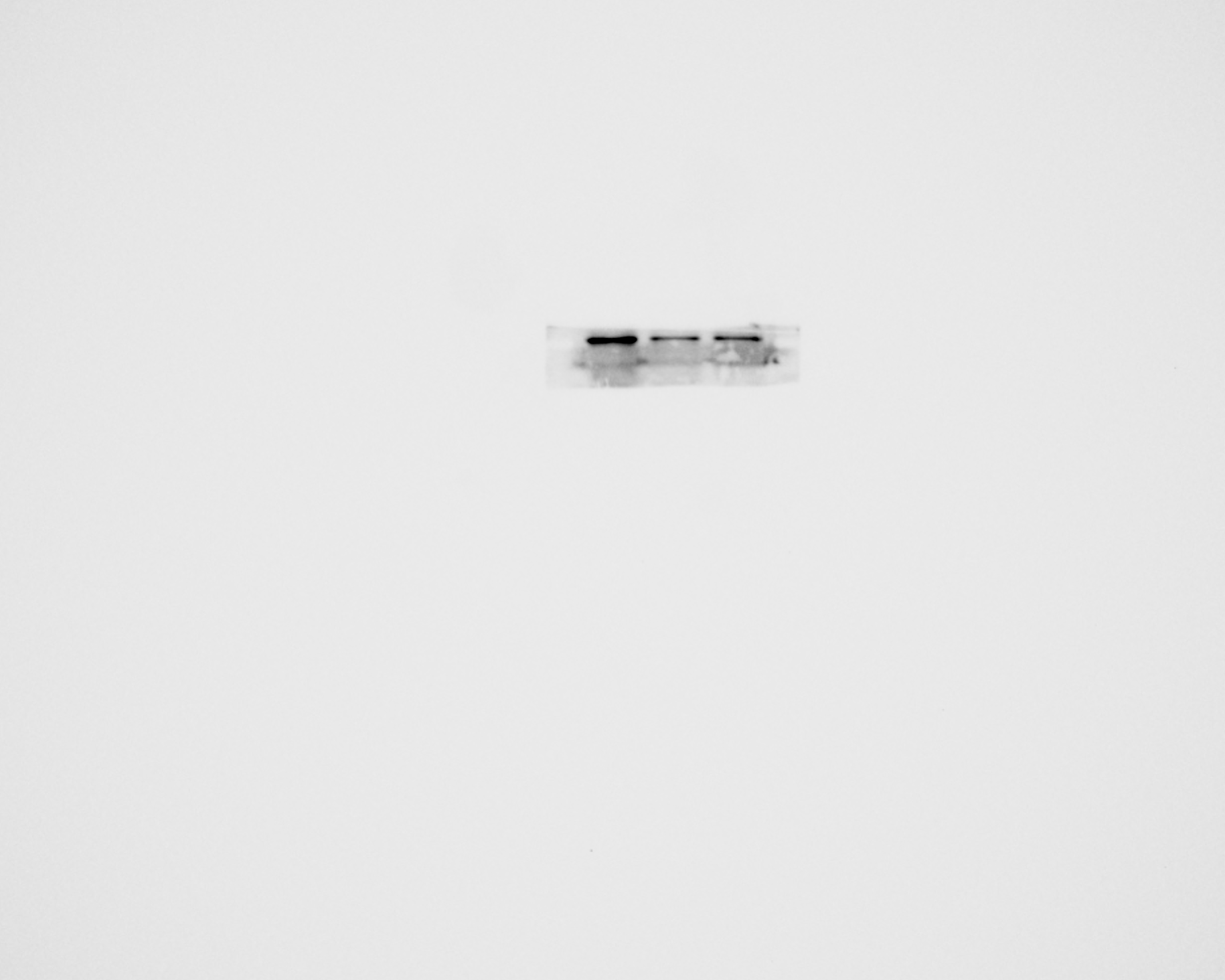

Supplement: Supplementary file 1 [file cancers-14-04151-s001.zip › File S1/Figure S3/FigerS3B SCC47 PRKCZ.tif]

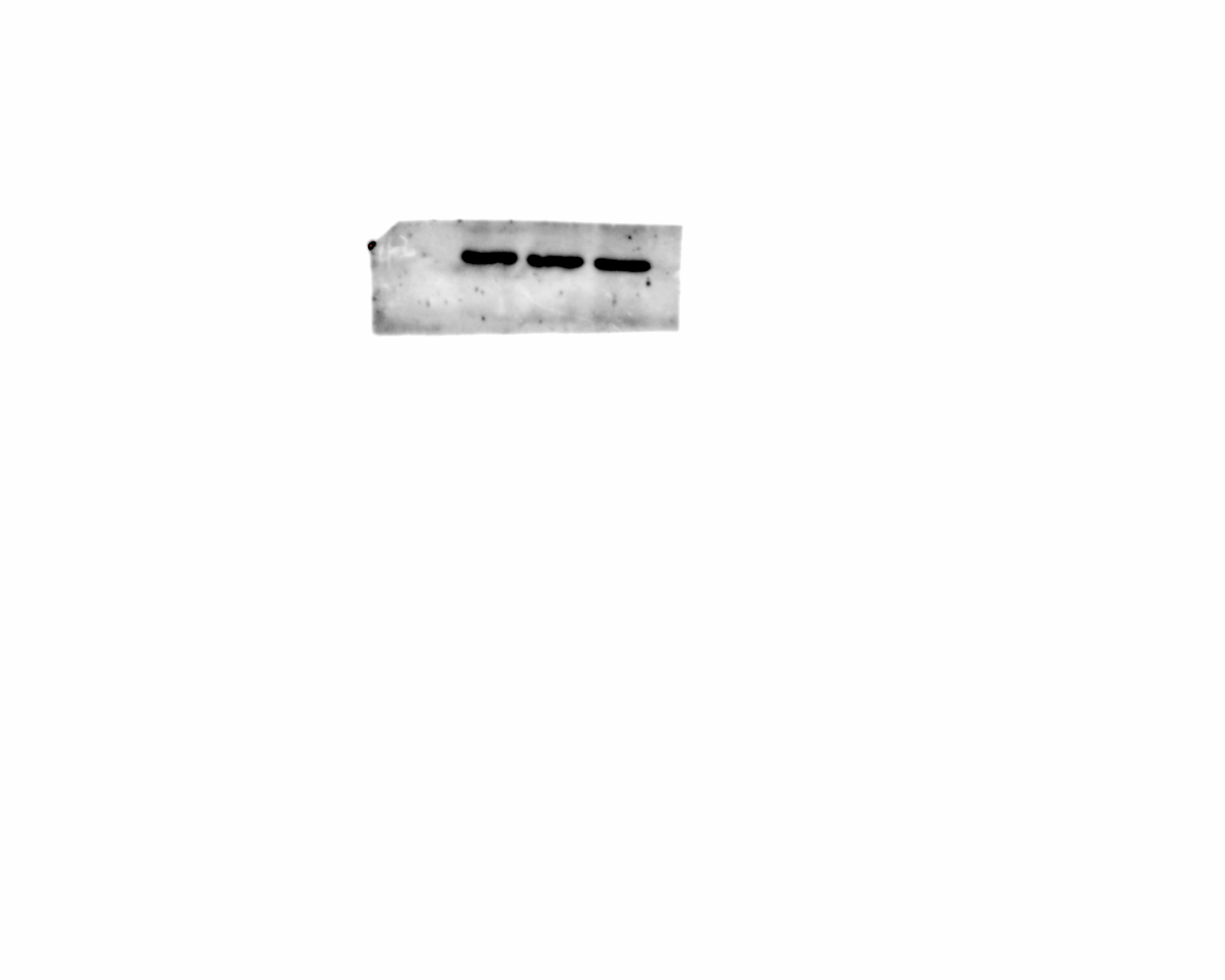

Supplement: Supplementary file 1 [file cancers-14-04151-s001.zip › File S1/Figure S3/FigerS3B Cal27+E6E7 gap.jpg]

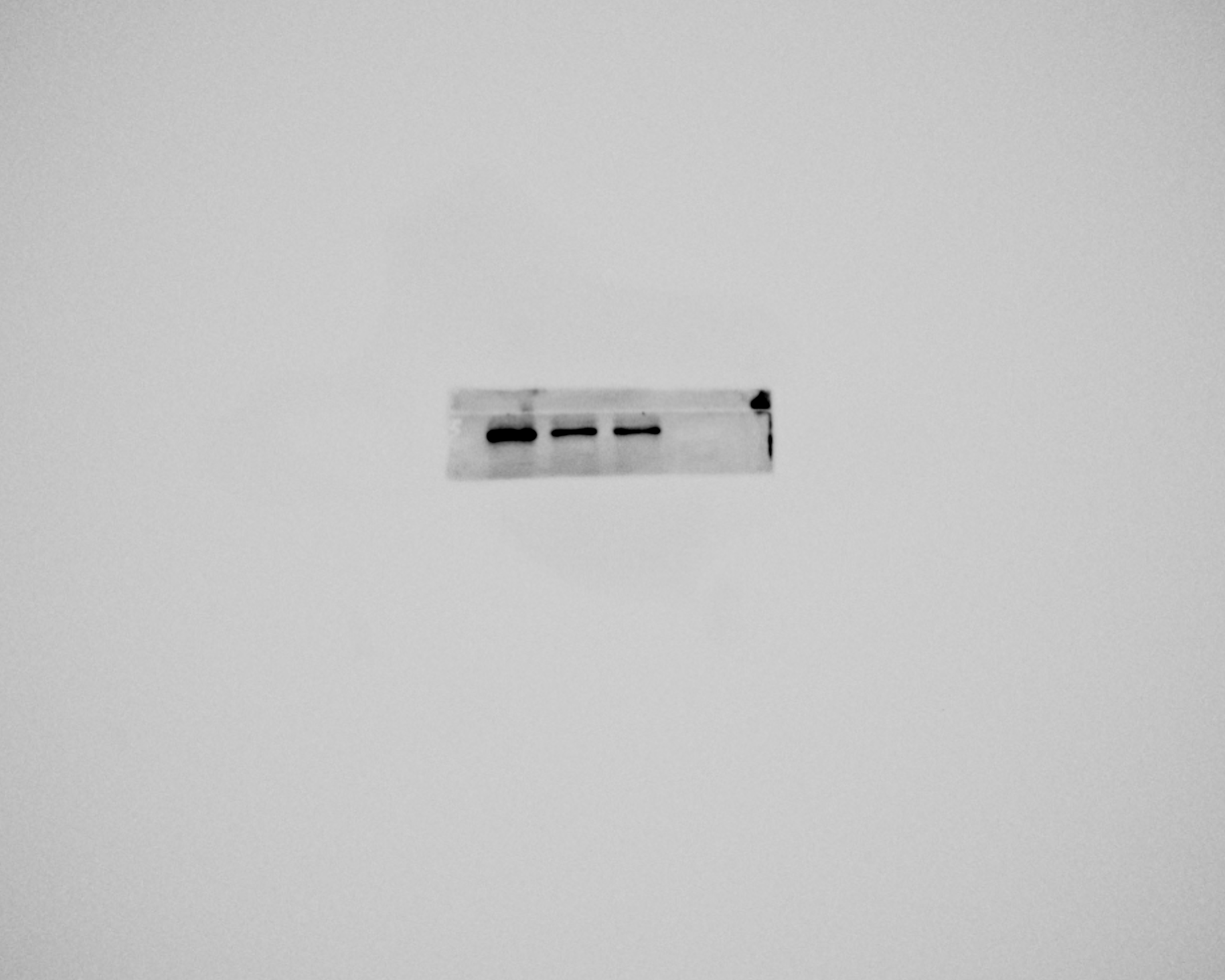

Supplement: Supplementary file 1 [file cancers-14-04151-s001.zip › File S1/Figure S3/FigerS3B Cal27+E6E7 prkcz.tif]

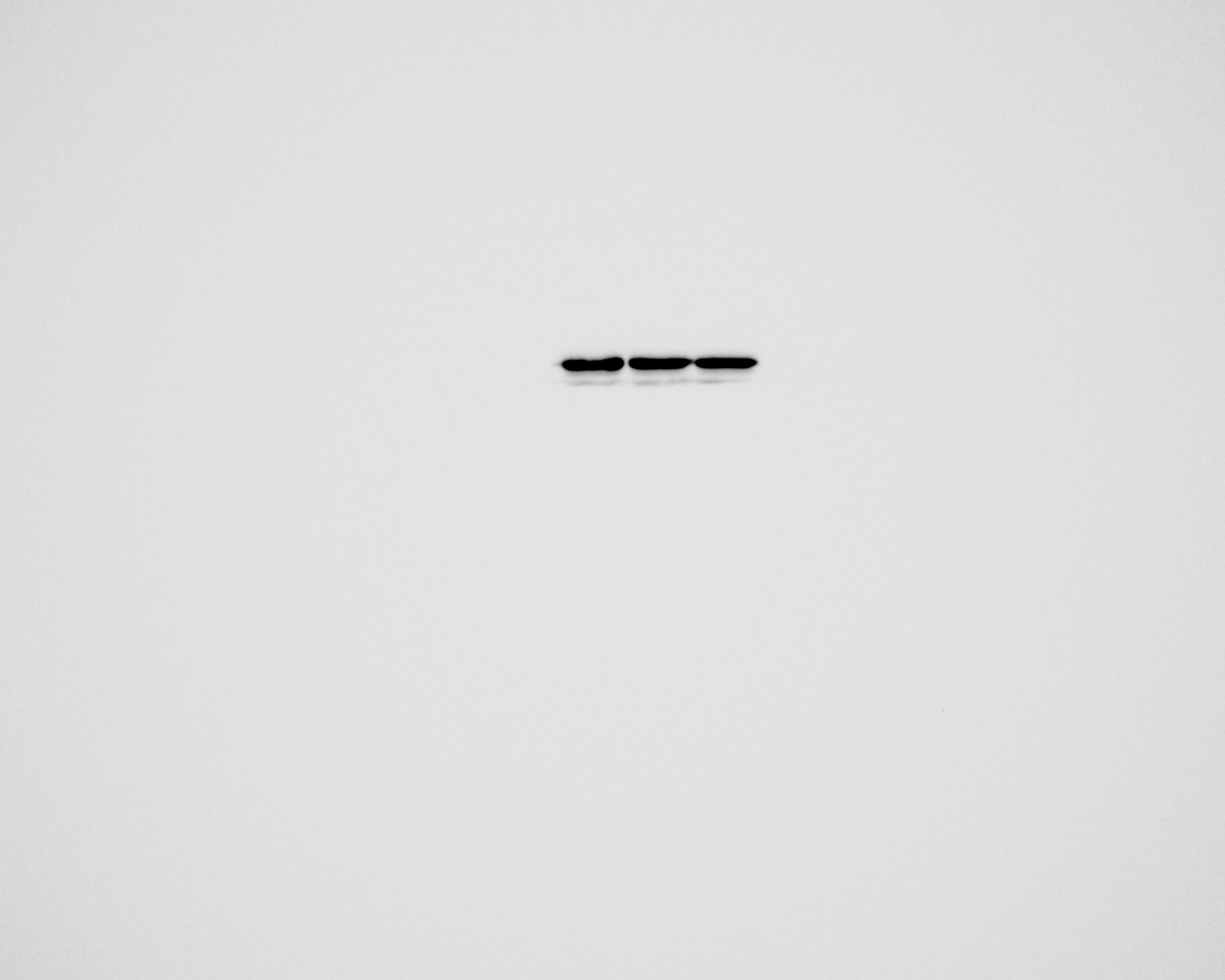

Supplement: Supplementary file 1 [file cancers-14-04151-s001.zip › File S1/Figure S3/FigerS3B SCC47 gap.jpg]

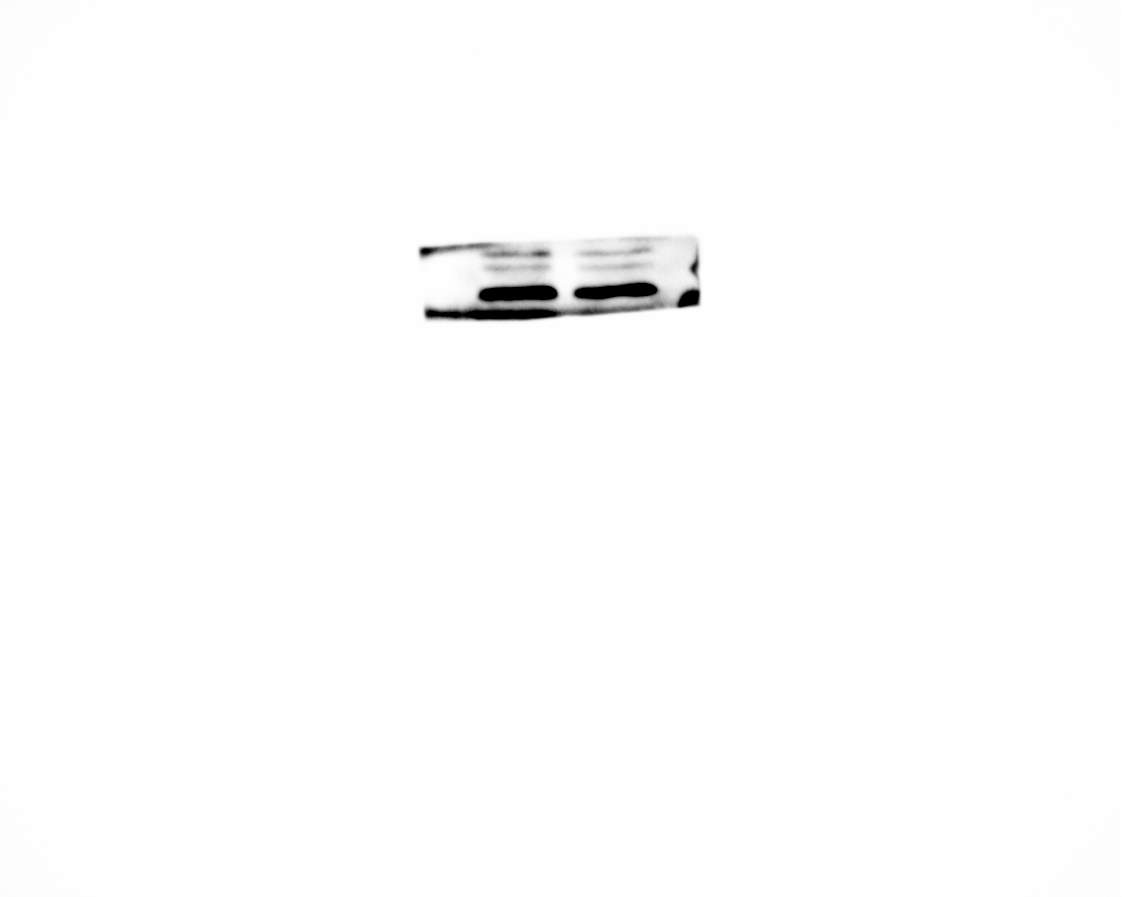

Supplement: Supplementary file 1 [file cancers-14-04151-s001.zip › File S1/Figure S3/FigerS3G gapdh.tif]

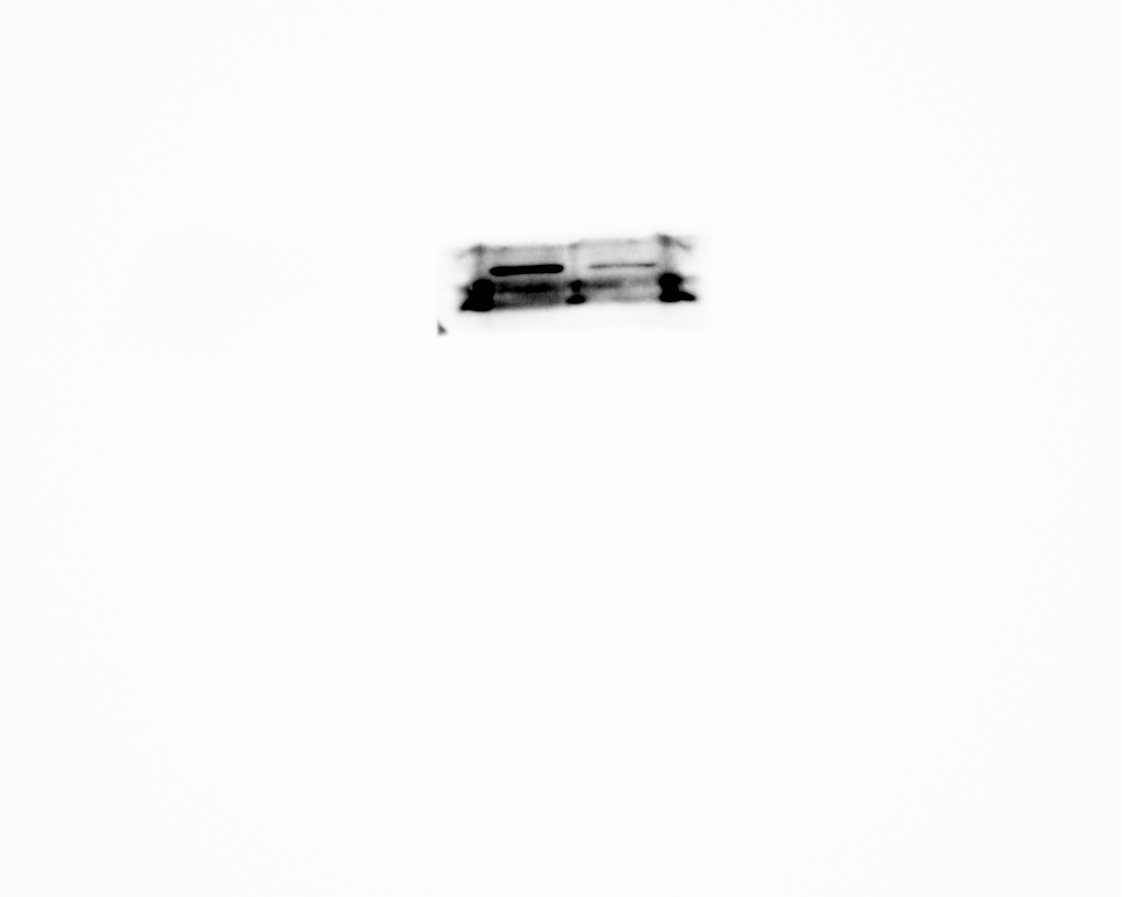

Supplement: Supplementary file 1 [file cancers-14-04151-s001.zip › File S1/Figure S3/FigerS3G PRKCZ.jpg]

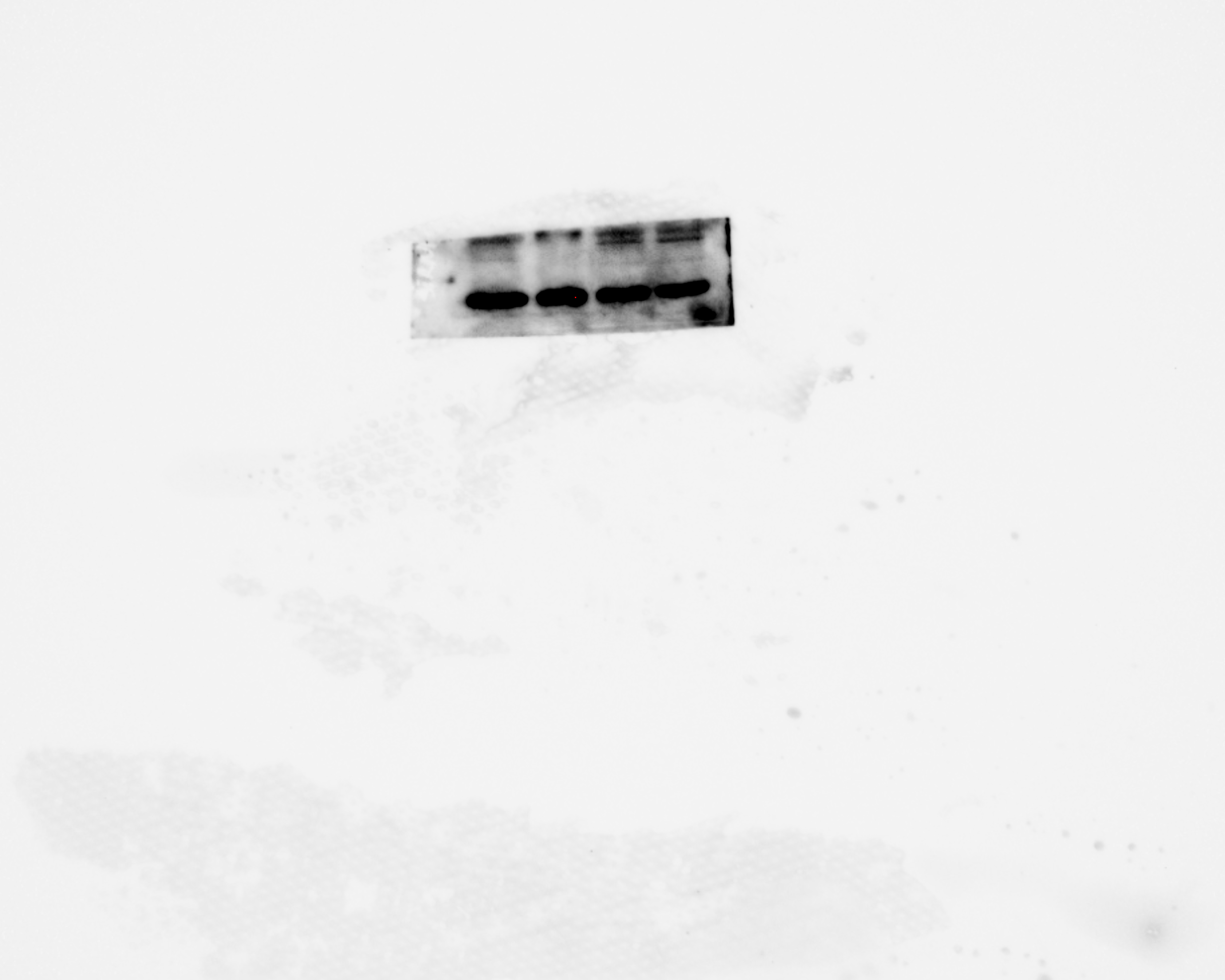

Supplement: Supplementary file 1 [file cancers-14-04151-s001.zip › File S1/Figure S4/Figure S4B siE6-Gapdh.tif]

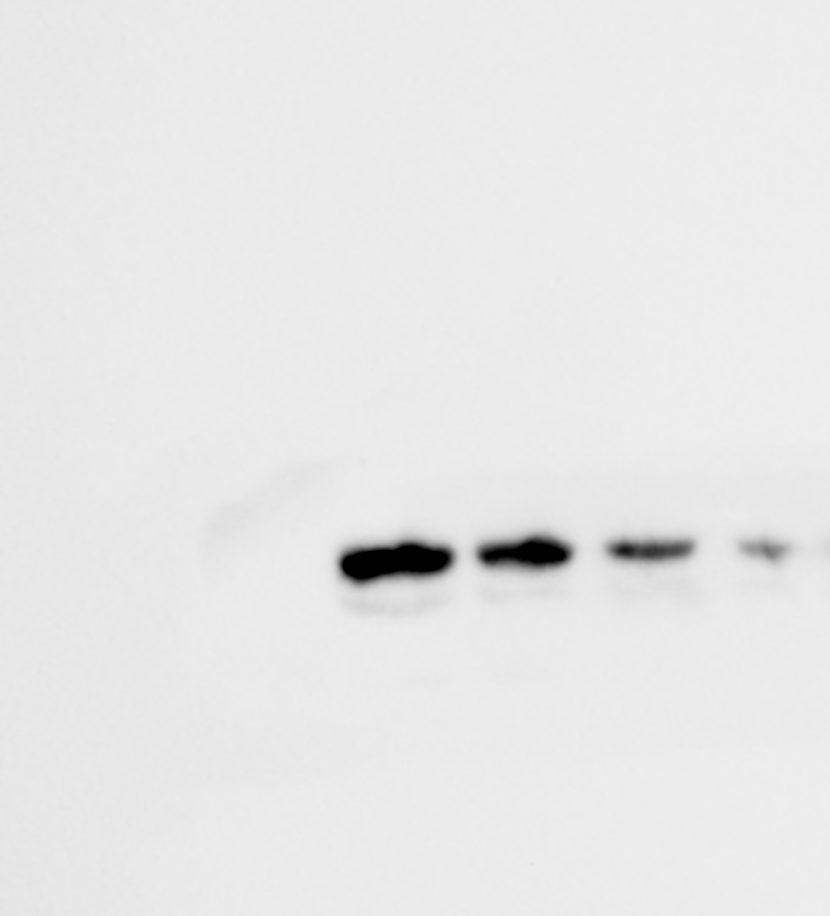

Supplement: Supplementary file 1 [file cancers-14-04151-s001.zip › File S1/Figure S4/Figure S4B siE6.tif]

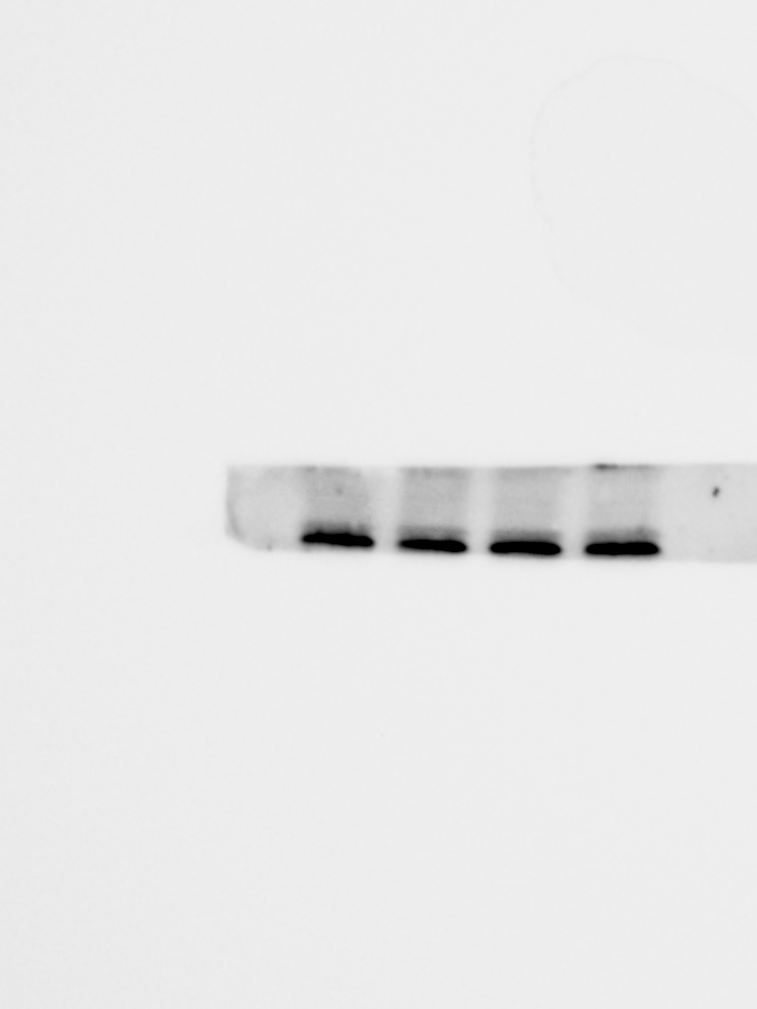

Supplement: Supplementary file 1 [file cancers-14-04151-s001.zip › File S1/Figure S4/Figure S4B siE7-Gapdh.tif]

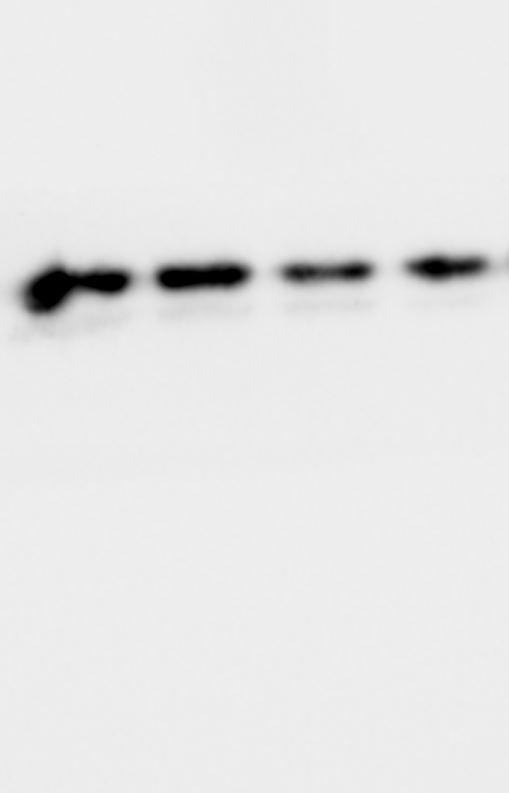

Supplement: Supplementary file 1 [file cancers-14-04151-s001.zip › File S1/Figure S4/Figure S4B siE7.tif]

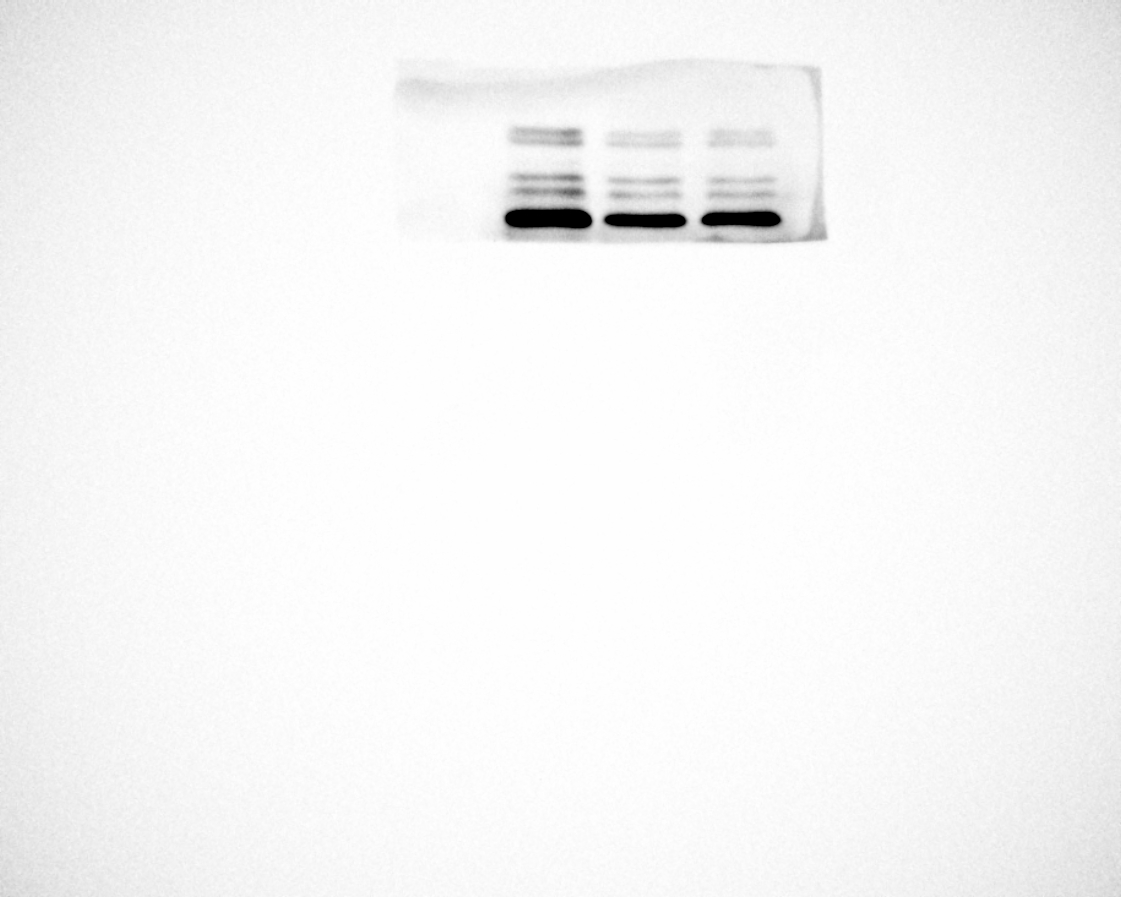

Supplement: Supplementary file 1 [file cancers-14-04151-s001.zip › File S1/Figure S4/Figure S4D DNMT1.jpg]

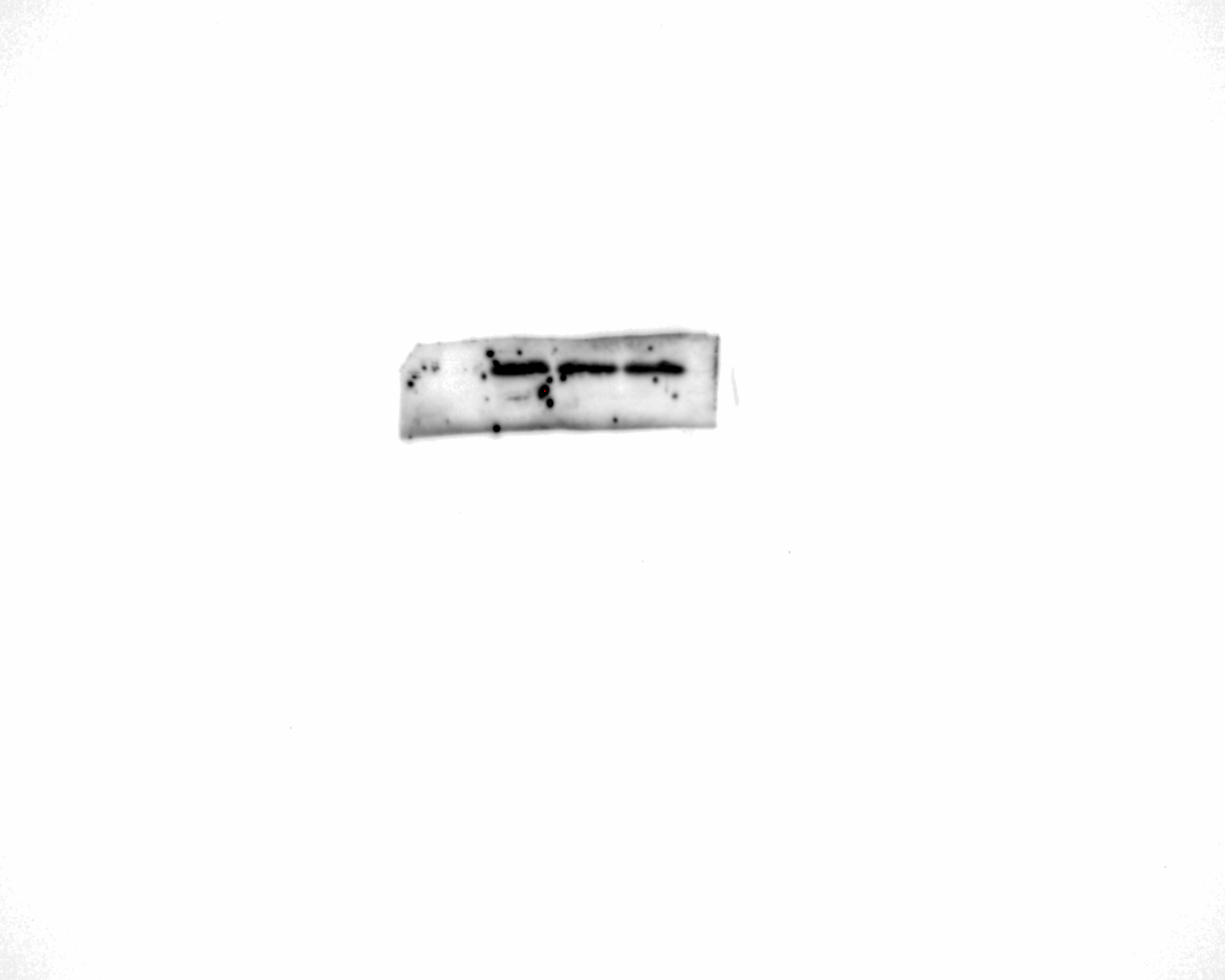

Supplement: Supplementary file 1 [file cancers-14-04151-s001.zip › File S1/Figure S4/Figure S4D DNMT3a.tif]

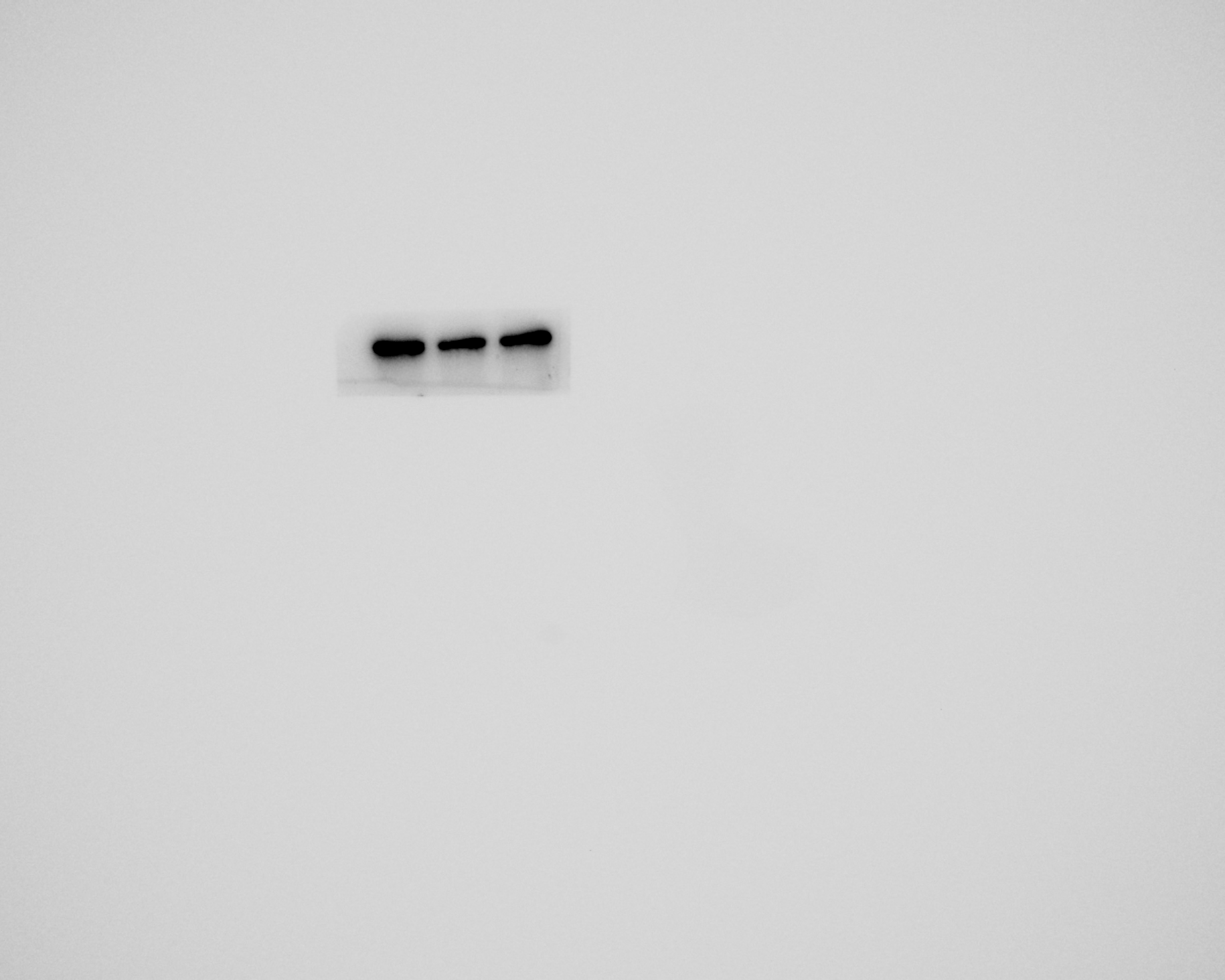

Supplement: Supplementary file 1 [file cancers-14-04151-s001.zip › File S1/Figure S4/Figure S4D DNMT3B.tif]

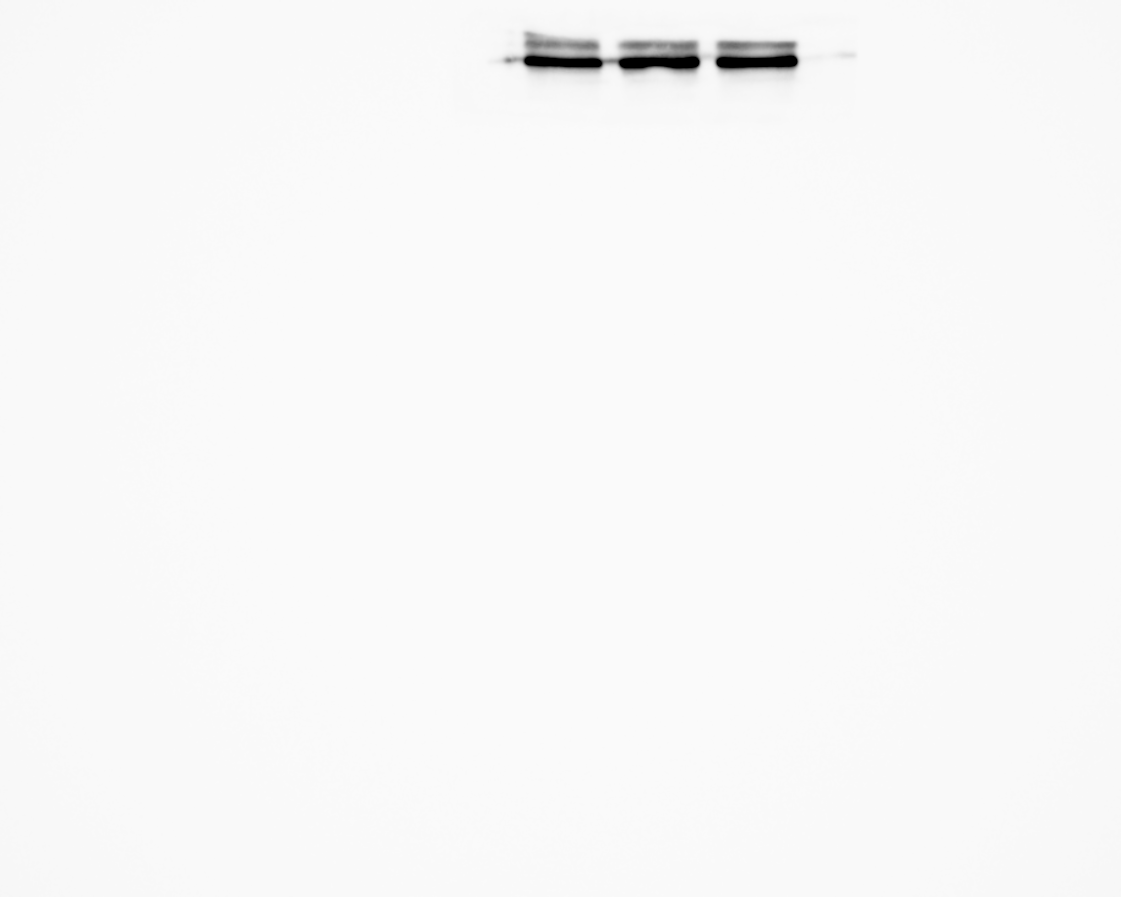

Supplement: Supplementary file 1 [file cancers-14-04151-s001.zip › File S1/Figure S4/Figure S4D Gapdh.tif]

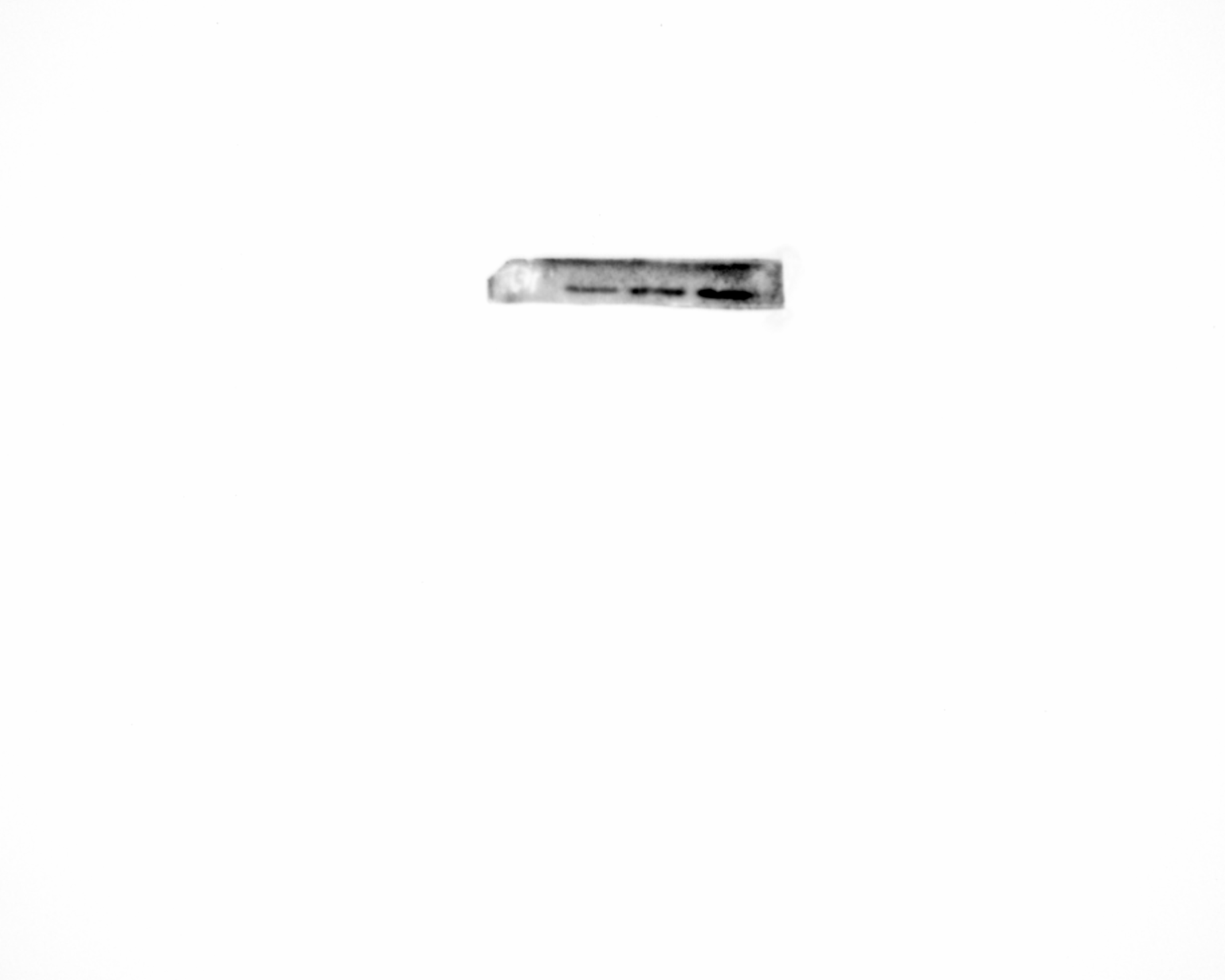

Supplement: Supplementary file 1 [file cancers-14-04151-s001.zip › File S1/Figure S5/FigerS5C Cal27+E6E7 cdc42.tif]

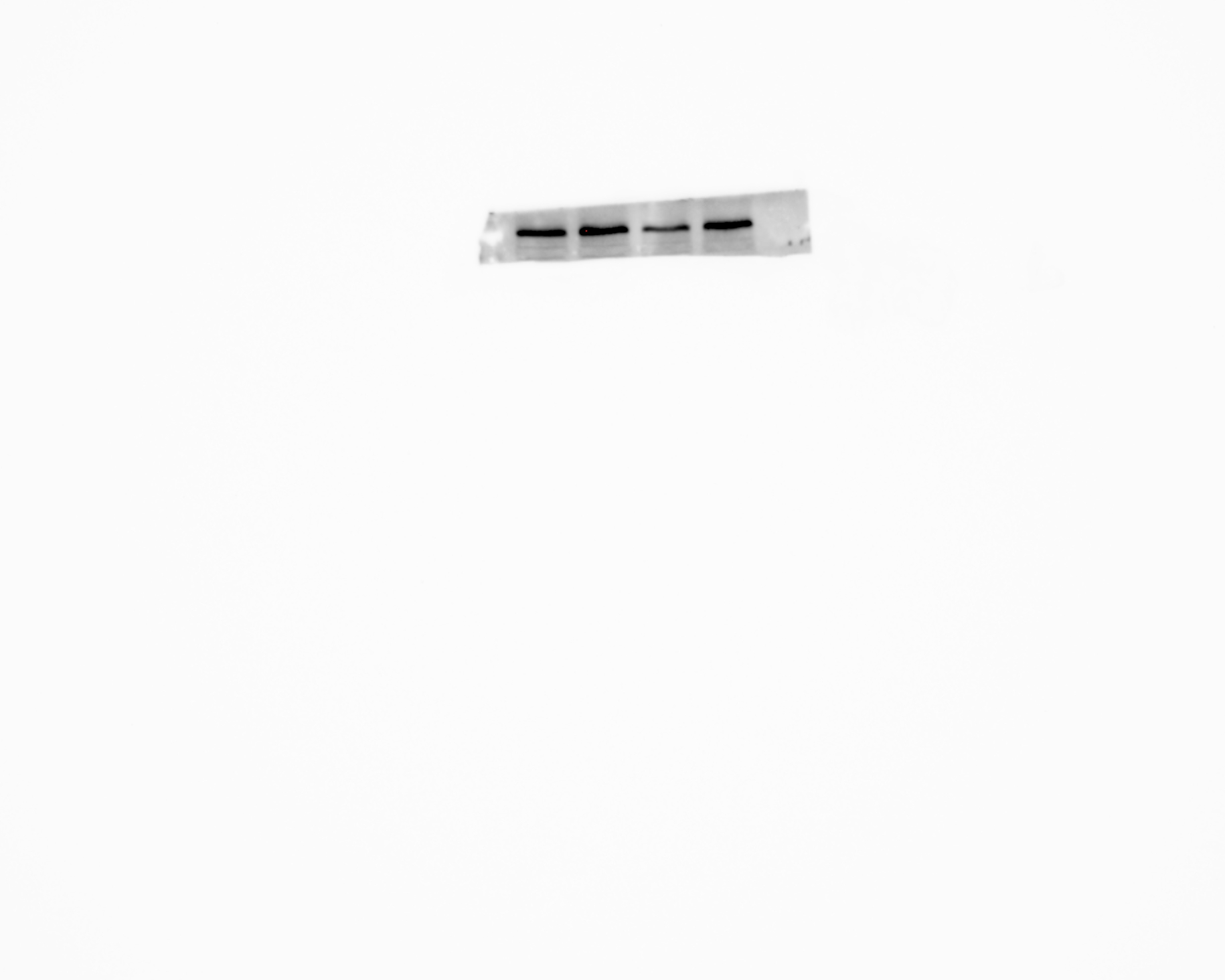

Supplement: Supplementary file 1 [file cancers-14-04151-s001.zip › File S1/Figure S5/FigerS5C Cal27+E6E7 PRKCZ.jpg]

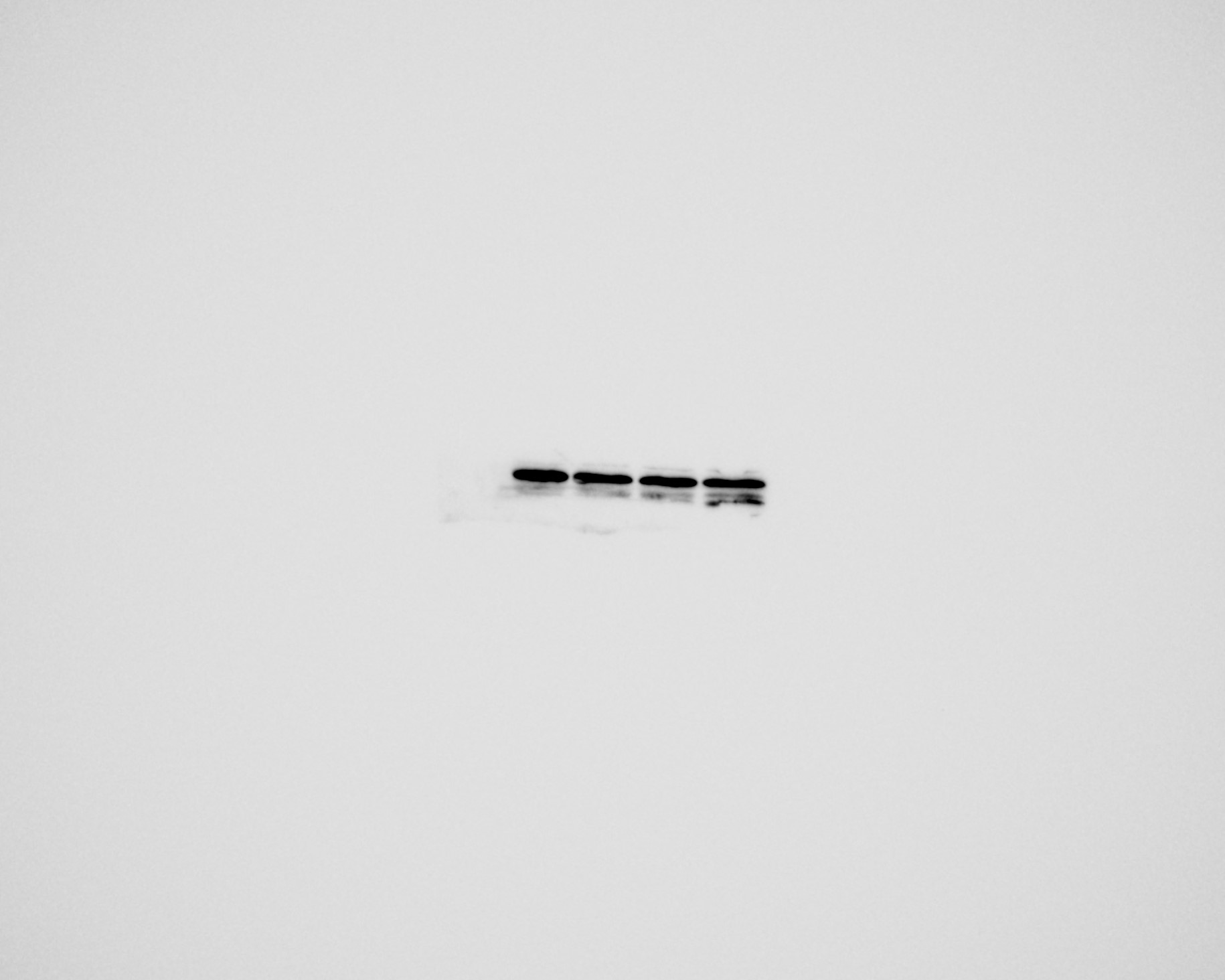

Supplement: Supplementary file 1 [file cancers-14-04151-s001.zip › File S1/Figure S5/FigerS5C Cal27+E6E7 GAP.jpg]

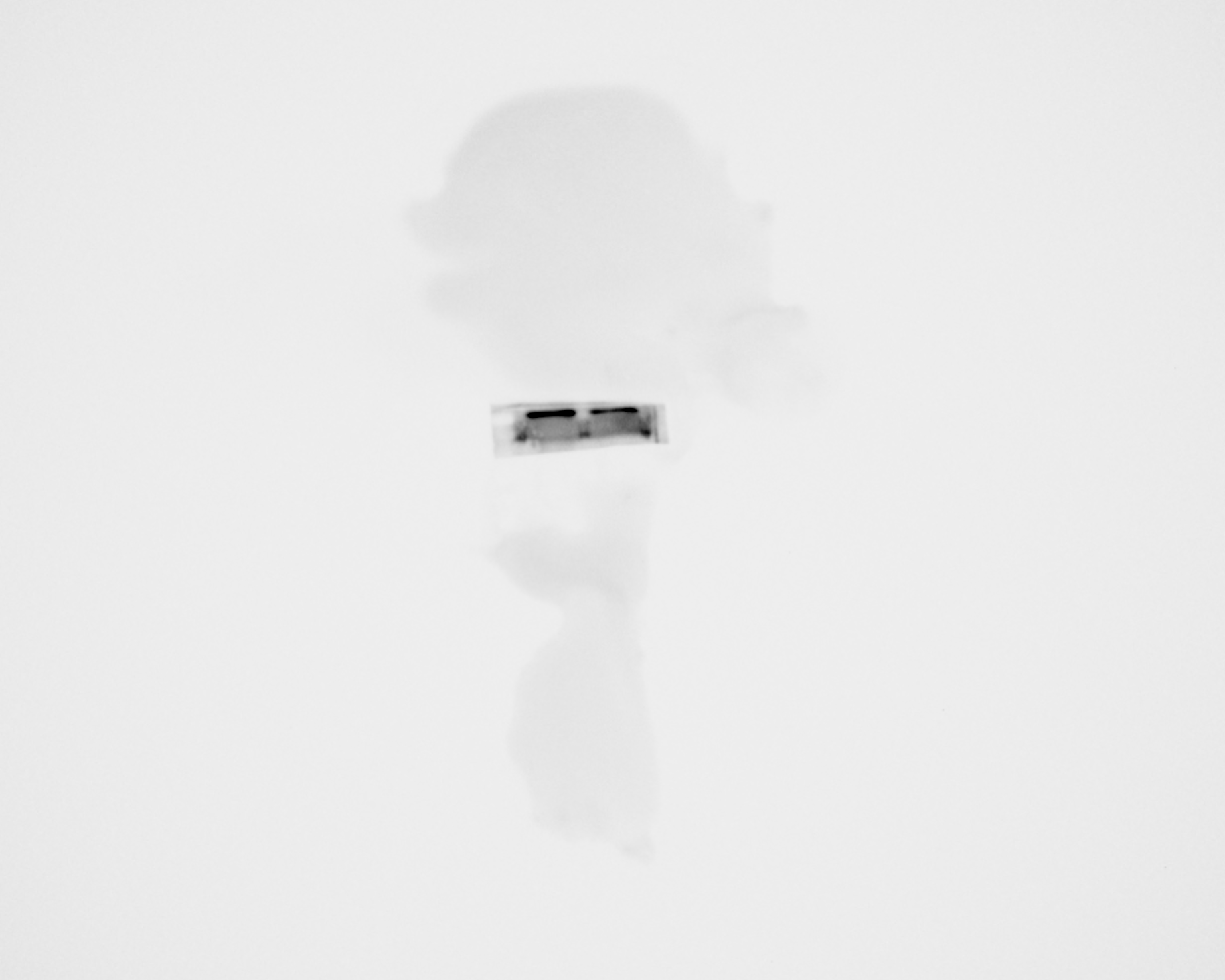

Supplement: Supplementary file 1 [file cancers-14-04151-s001.zip › File S1/Figure S5/FigerS5C SCC47 cdc42.tif]

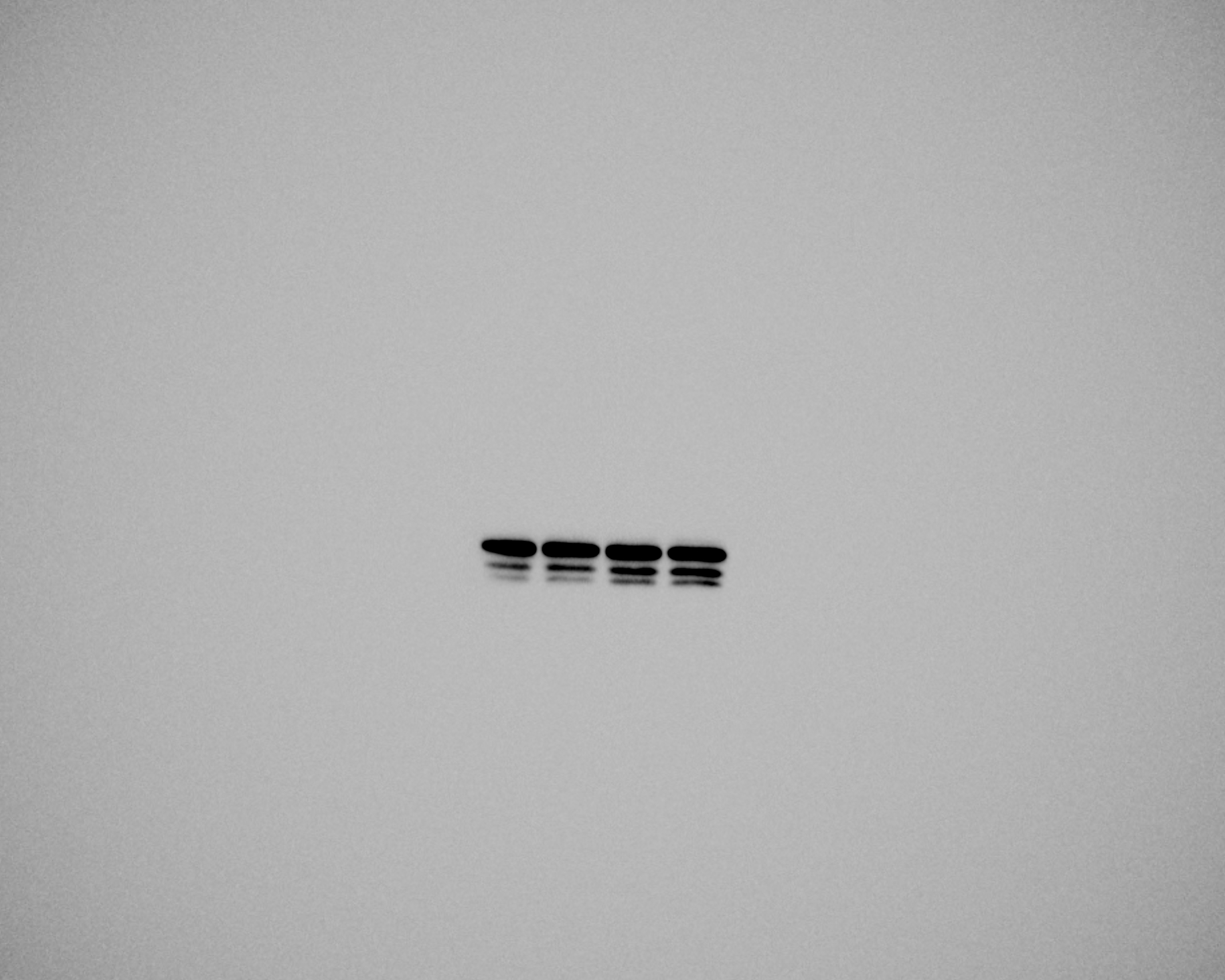

Supplement: Supplementary file 1 [file cancers-14-04151-s001.zip › File S1/Figure S5/FigerS5C SCC47 gap.jpg]

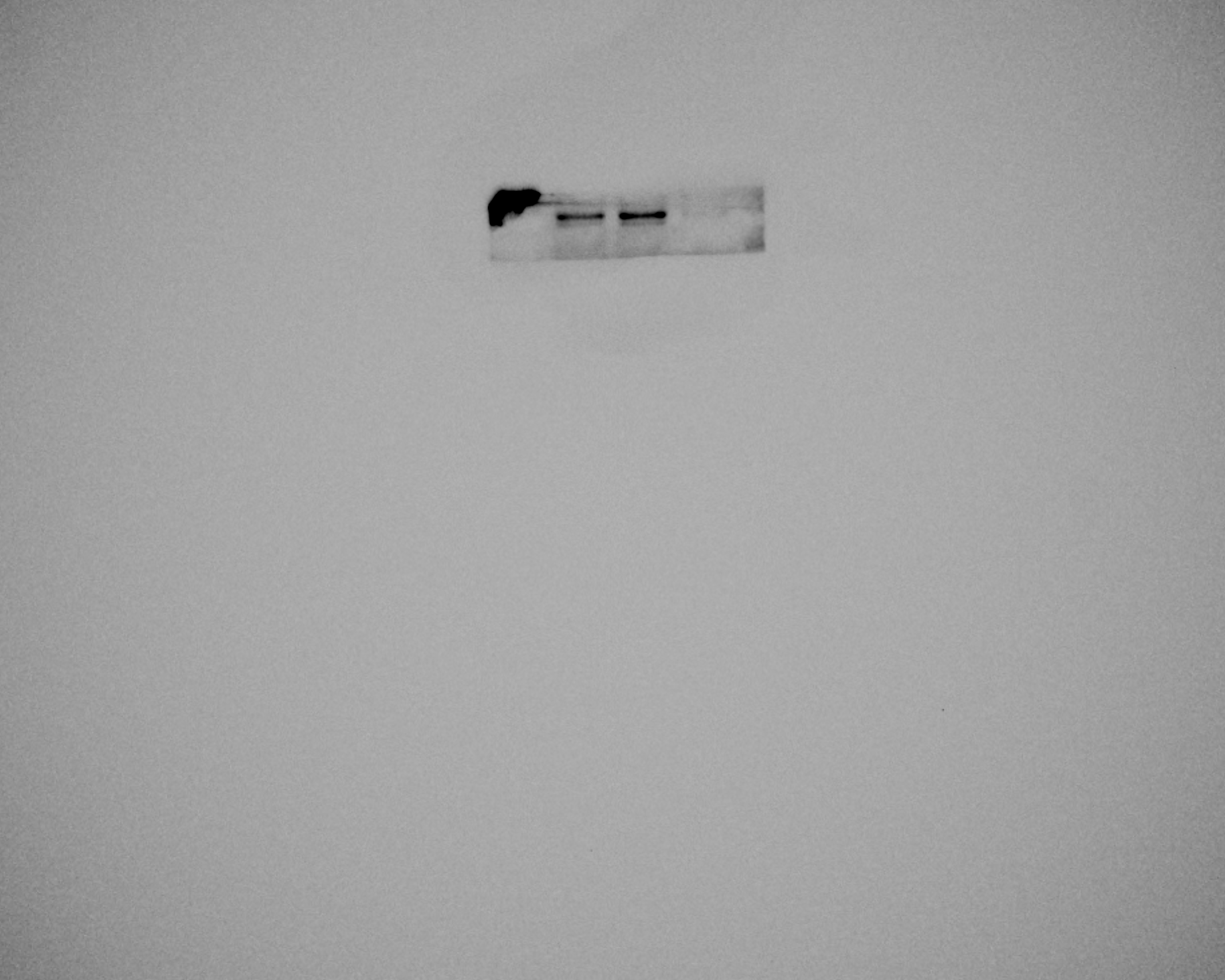

Supplement: Supplementary file 1 [file cancers-14-04151-s001.zip › File S1/Figure S5/FigerS5C SCC47 PRKCZ.tif]

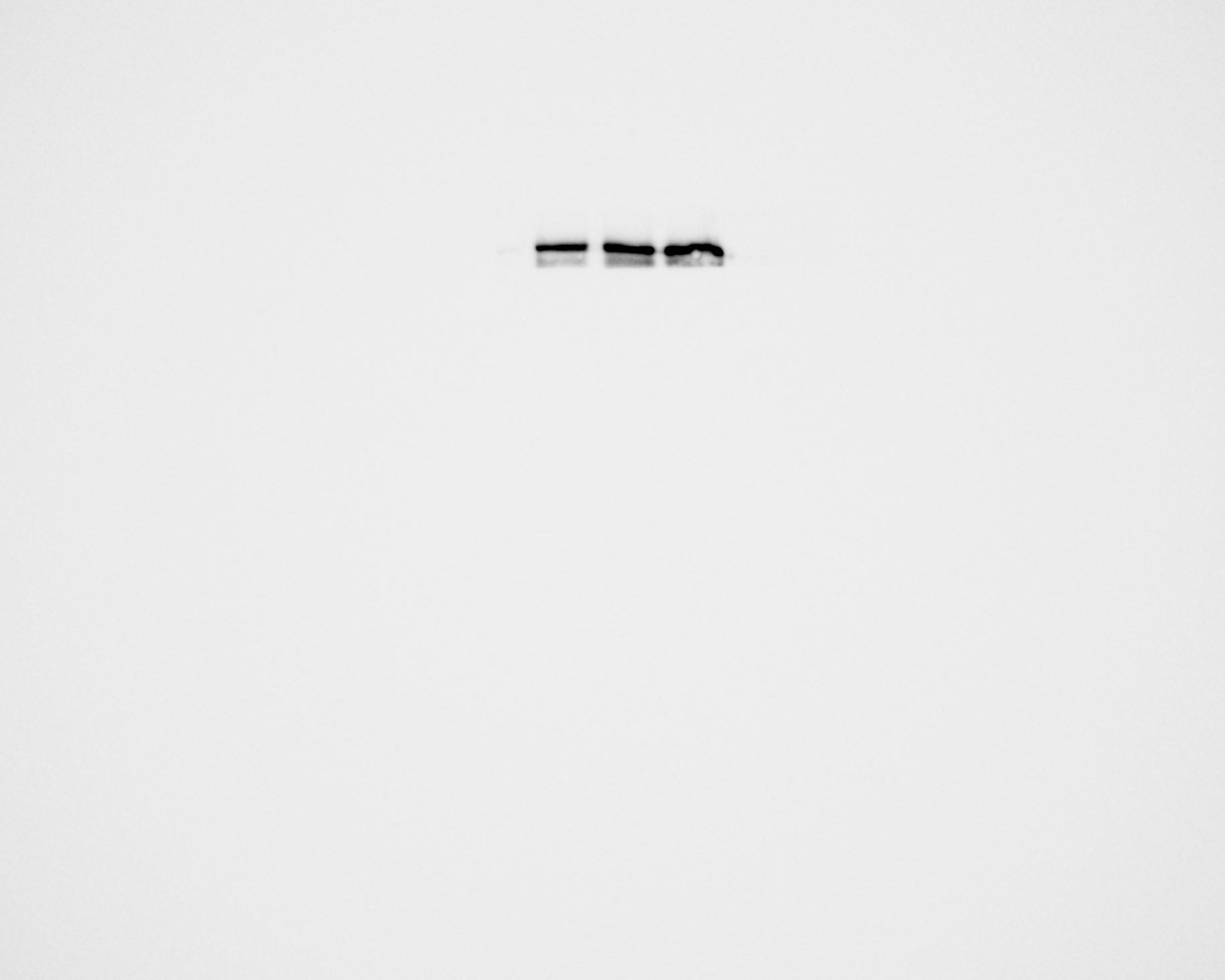

Supplement: Supplementary file 1 [file cancers-14-04151-s001.zip › File S1/Figure S5/FigerS5D Cal27+E6E7 ecad.jpg]

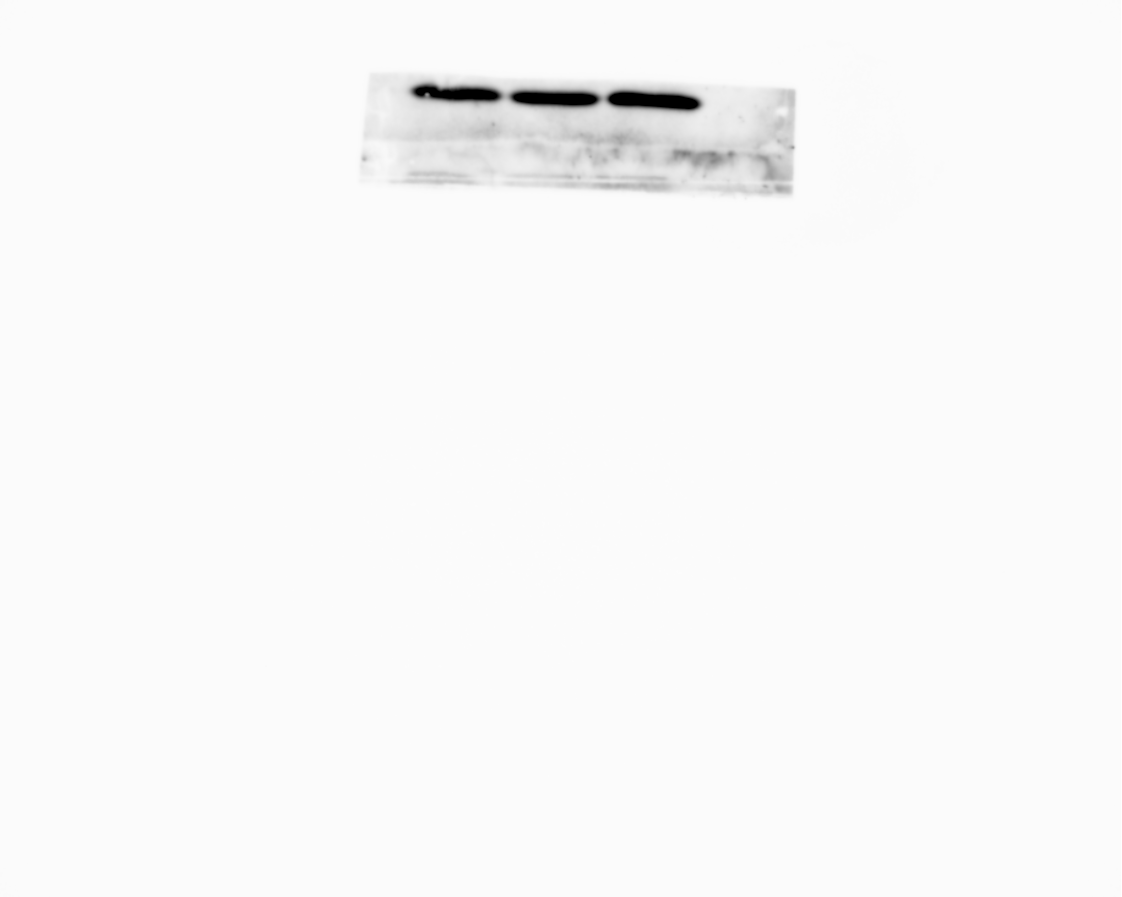

Supplement: Supplementary file 1 [file cancers-14-04151-s001.zip › File S1/Figure S5/FigerS5D Cal27+E6E7 gap.tif]

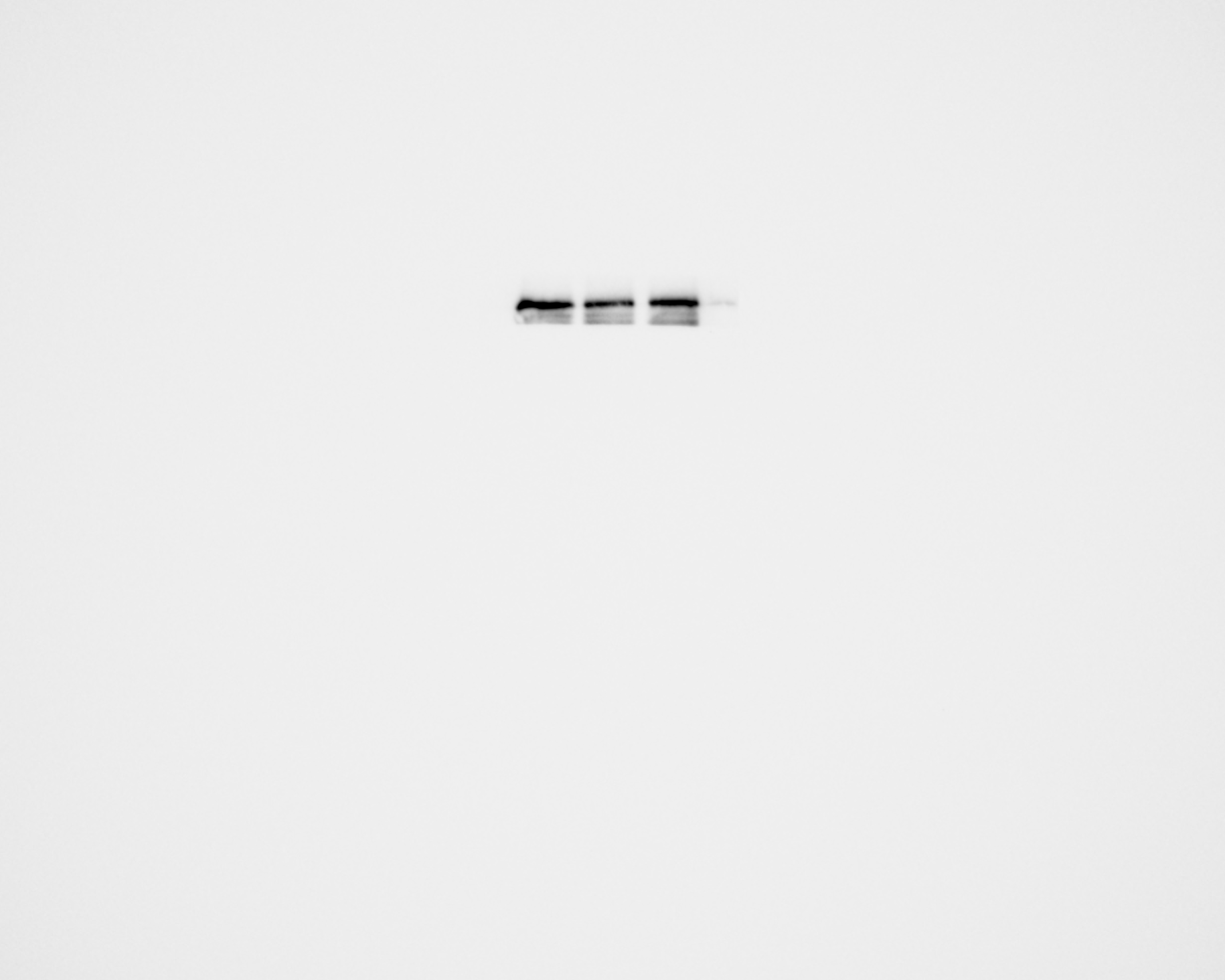

Supplement: Supplementary file 1 [file cancers-14-04151-s001.zip › File S1/Figure S5/FigerS5D Cal27+E6E7 ncad.tif]

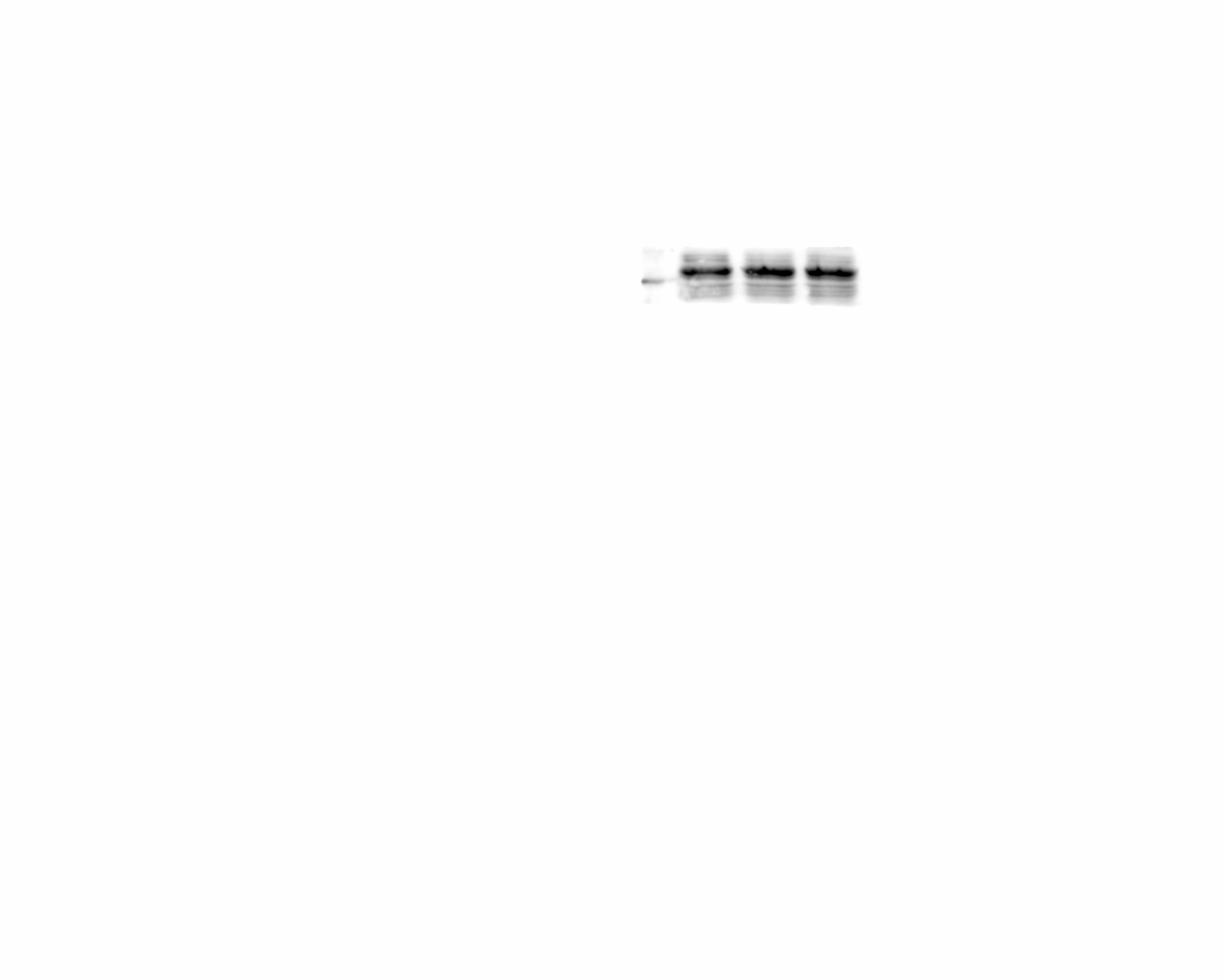

Supplement: Supplementary file 1 [file cancers-14-04151-s001.zip › File S1/Figure S5/FigerS5D Cal27+E6E7 VIM.tif]

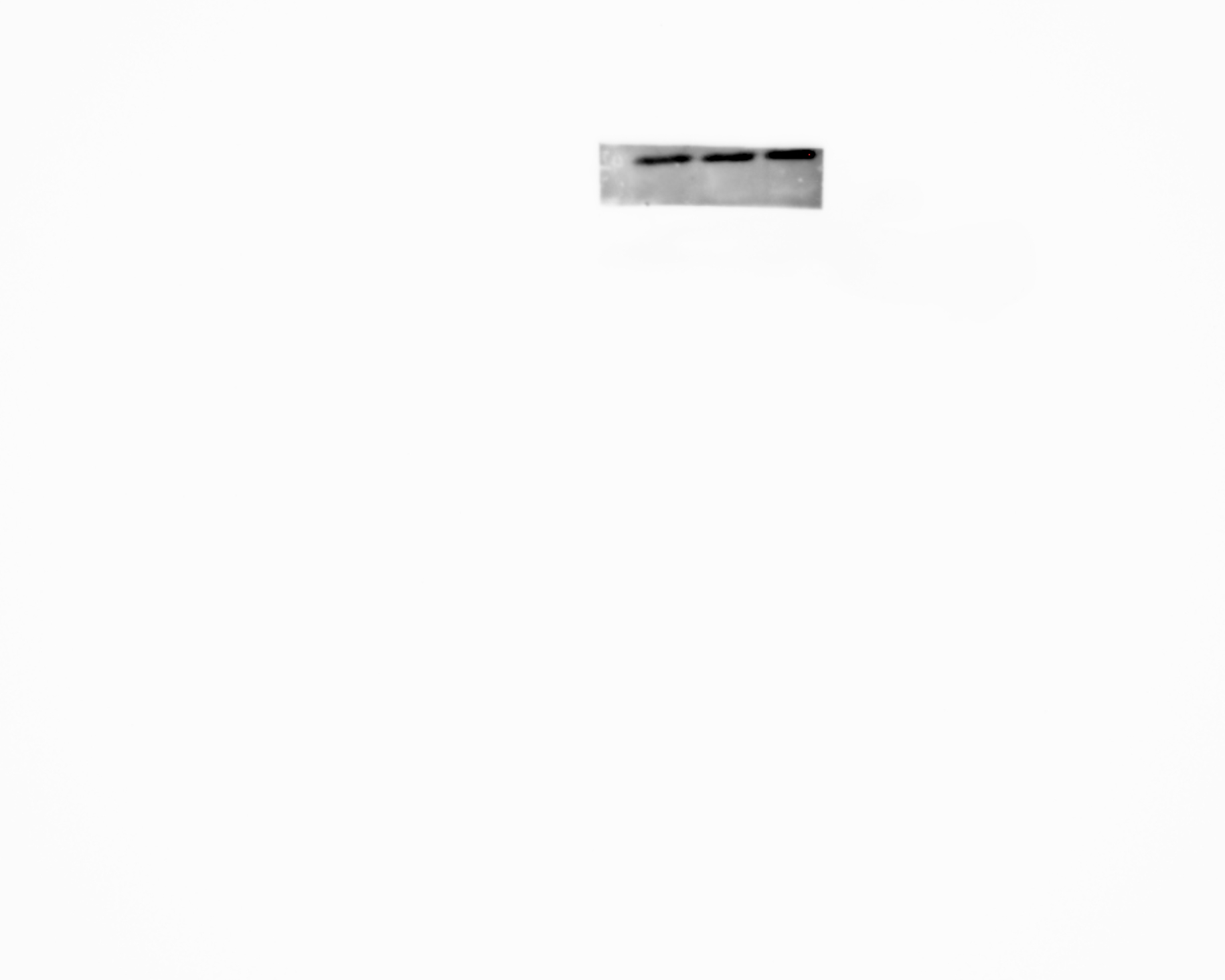

Supplement: Supplementary file 1 [file cancers-14-04151-s001.zip › File S1/Figure S5/FigerS5D SCC47 ecad.jpg]

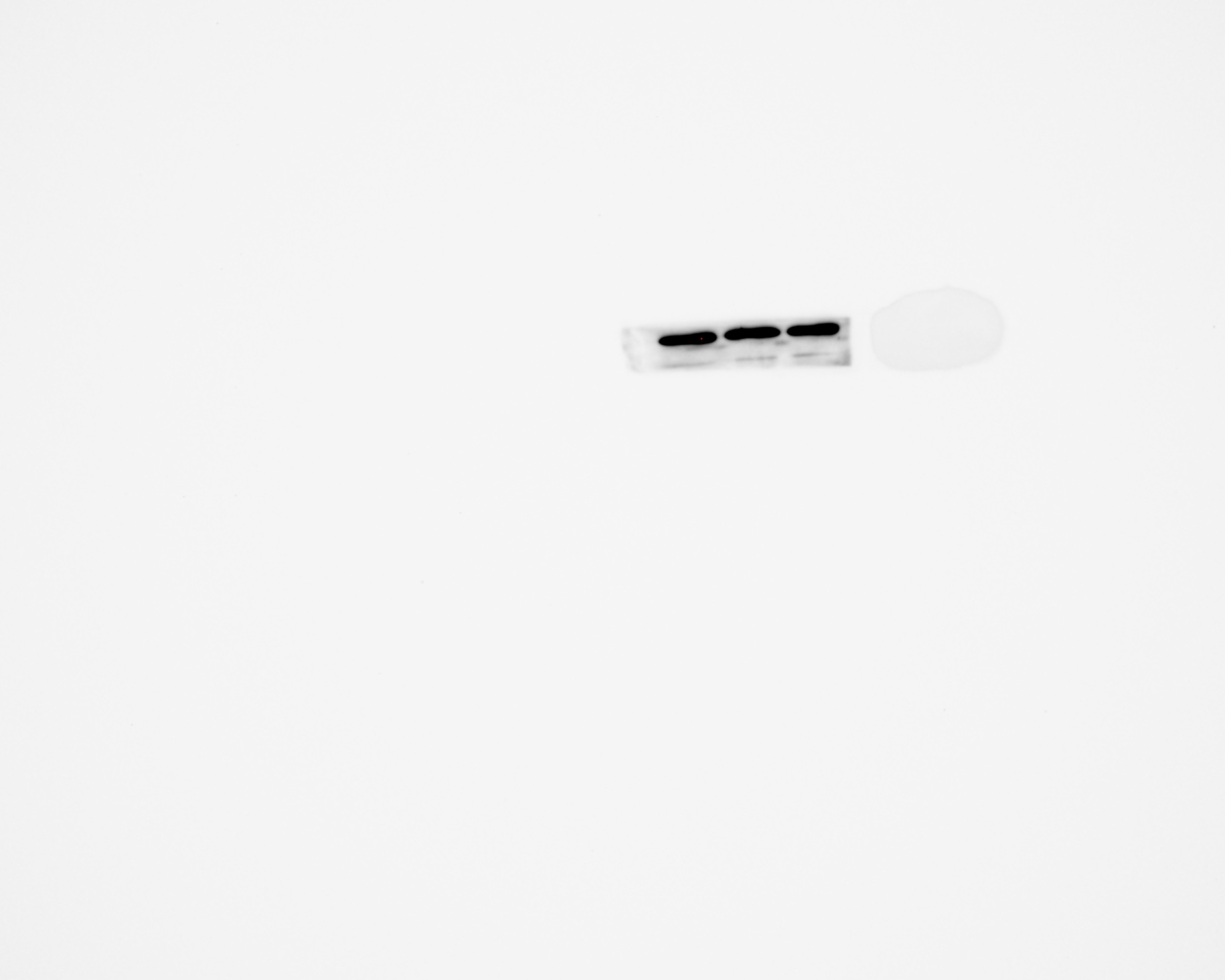

Supplement: Supplementary file 1 [file cancers-14-04151-s001.zip › File S1/Figure S5/FigerS5D SCC47 gap.jpg]

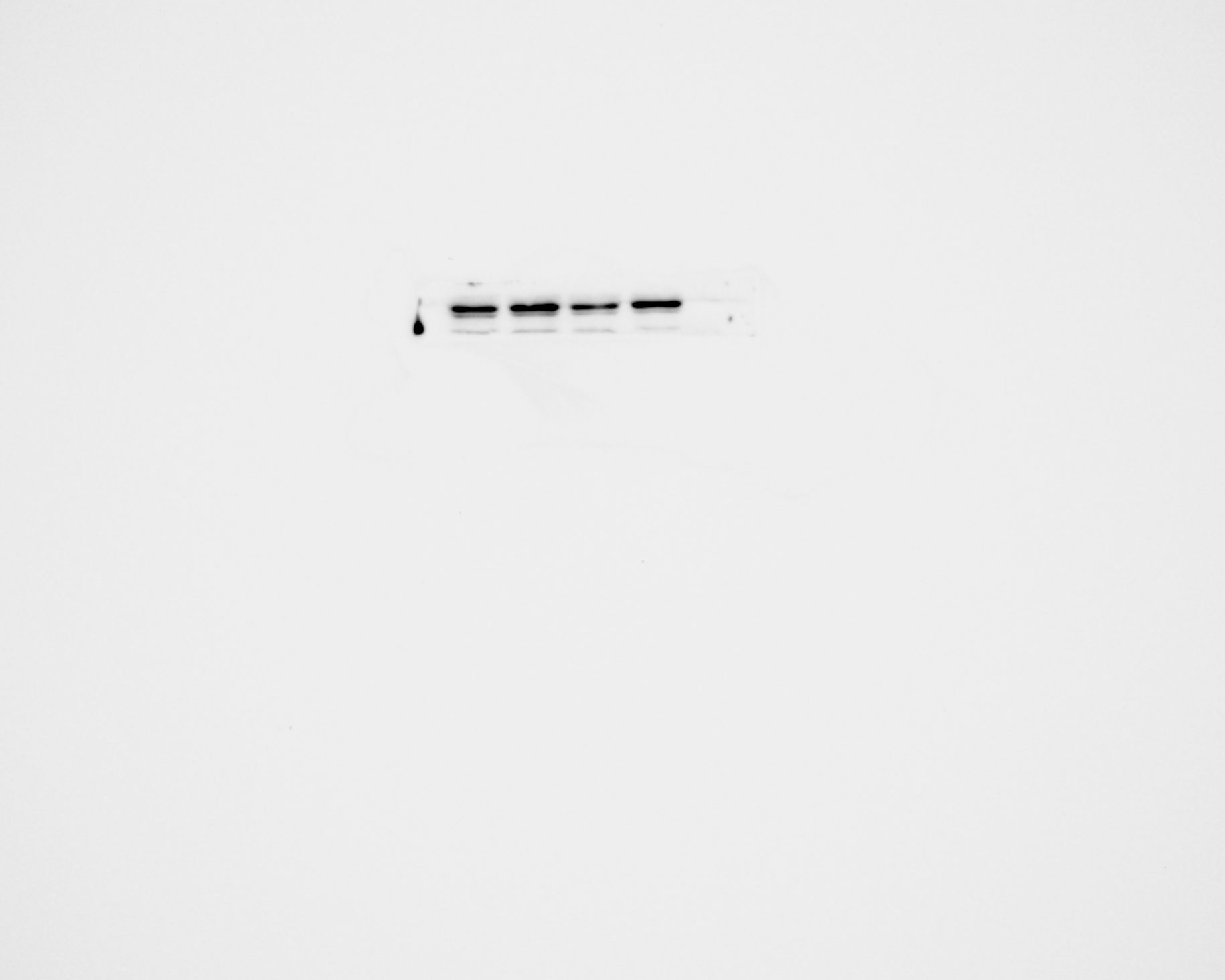

Supplement: Supplementary file 1 [file cancers-14-04151-s001.zip › File S1/Figure S5/FigerS5D SCC47 ncad.tif]

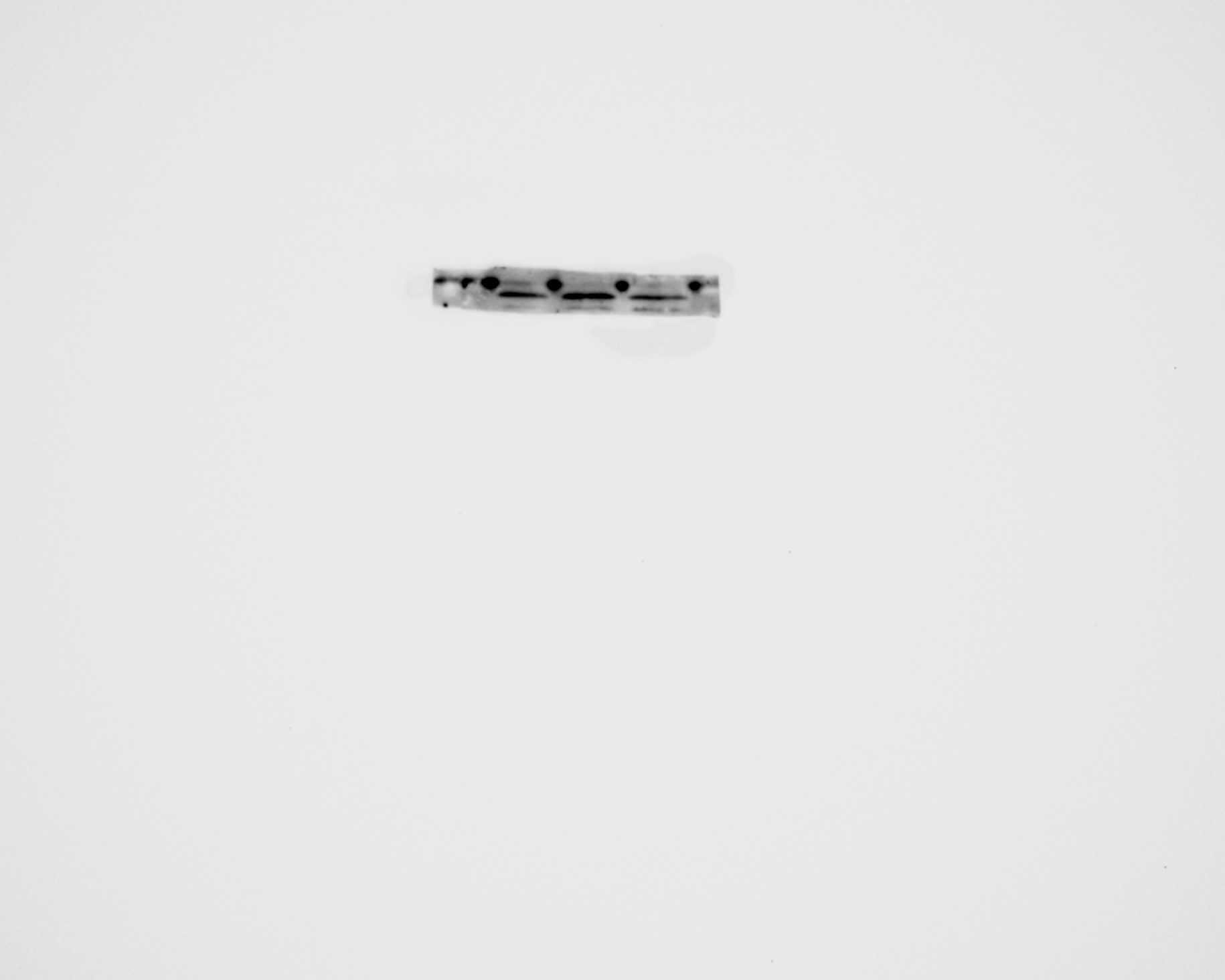

Supplement: Supplementary file 1 [file cancers-14-04151-s001.zip › File S1/Figure S5/FigerS5D SCC47 vim.jpg]
